# Strain Partitioning in a Flattening Shear Zone: Re‐Evaluation of a Cycladic Style Detachment

**DOI:** 10.1029/2024TC008412

**Published:** 2024-11-21

**Authors:** T. A. Ducharme, D. A. Schneider, B. Grasemann, V. Scoging, C. Bakowsky, K. P. Larson, A. Camacho

**Affiliations:** ^1^ Department of Earth and Environmental Science University of Ottawa Ottawa ON Canada; ^2^ Now at Département de Géologie et de Génie Géologique Université Laval Québec QC Canada; ^3^ Department of Geology University of Vienna Vienna Austria; ^4^ Department of Earth, Environmental and Geographic Sciences University of British Columbia Okanagan Kelowna BC Canada; ^5^ Department of Earth Sciences University of Manitoba Winnipeg MB Canada

**Keywords:** ductile thinning, cycladic blueschist unit, exhumation, detachment faulting, geochronology, Greece

## Abstract

The Almyropotamos tectonic window on southern Evia island in the NW Aegean Sea divides two high pressure‐low temperature metamorphic units, representing distinct Hellenic thrust sheets. Ductile thinning along the major low‐angle Evia Shear Zone has closely juxtaposed the lower (Basal Unit) marble‐flysch sequence structurally below Styra marbles (Cycladic Blueschist Unit). The partially attenuated flysch comprises a matrix dominated by pelitic schist, with dispersed cm‐ to hm‐scale blocks of marble, carbonate schist, quartzite, and metabasite. Structural investigation of the different lithotypes in the flysch reveals tectonic fabrics related to general flattening strain are developed most strongly in the pelitic matrix, whereas the compositionally diverse blocks exhibit differential preservation of older structures. Quartz c‐axis distributions from quartz veins in the schists reflect an early, moderate temperature plane strain deformation. Colder deformation is evident in some pelitic schists, capturing Z‐centered girdles consistent with the oblate finite strain ellipsoid inferred from macroscopic structures. New in situ ^40^Ar/^39^Ar and ^87^Rb/^87^Sr geochronology delineate the timing of the two deformation events. Geochronological data reaffirm the first‐order observations of strain partitioning behavior at the scale of the shear zone, and confirm that the structure records two resolvable tectonometamorphic events: an early Oligocene HP‐LT event, and a late Oligocene‐early Miocene greenschist facies overprint coinciding with ductile thinning. The diffuse and discontinuous style of deformation recorded within the shear zone is unusual for major structures facilitating exhumation in the Aegean Sea, and may represent an analogue to mélange‐hosted shear zones that accommodate progressive strain during subduction.

## Key Points

- In situ white mica geochronology and structural data support a two‐stage deformation history for the Evia Shear Zone
- Differential preservation of structures and white mica dates are controlled by lithological heterogeneities in syn‐tectonic flysch
- Finite strain does not systematically intensify toward the hanging wall, perhaps indicating atypical detachment architecture

## 1 Introduction

From their first recognition in the rock record more than 40 years ago, examination of low‐angle normal faults (i.e., detachments) has revolutionized our understanding of the mechanics governing the return of deeply exhumed rocks to the surface (Lister & Davis, 1989; Lister et al., 1984; Scott & Lister, 1992; Wernicke, 1981; Wernicke & Axen, 1988). Detachment faulting is responsible for a significant magnitude of the unroofing observed in many young orogens, including the Basin and Range province, the Himalaya, and the Cyclades (Burchfiel et al., 1992; Carosi et al., 1998; Davis & Lister, 1988; Foster & John, 1999; Gautier & Brun, 1994; Grasemann et al., 2012; Jolivet et al., 2010). Regional‐scale structures of this type often impart a conspicuous and visually striking strain signature well into their footwalls, and create predictable structural relationships that readily permit their identification in the field (Jolivet et al., 2010; Lister & Davis, 1989). Nevertheless, detachment faults may evolve in diverse ways that are controlled by the geological and mechanical conditions of their surroundings during their lifespans (e.g., Braathen et al., 2004; Coleman et al., 2020; Davis et al., 2002; Kellett & Grujic, 2012; Krohe & Mposkos, 2002; Lecomte et al., 2010; Mehl et al., 2007).

Detachment faults are rarely the sole determinants of exhumation, with several other processes often contributing to the overall unroofing of a particular region (Froitzheim et al., 2003; Ring, Brandon, et al., 1999; Ring et al., 2010; Warren, 2013). For instance, wedge extrusion may be the process responsible for large magnitudes of exhumation at depth, whereas erosion, detachment faulting, and ductile thinning may dominate at shallower levels and control the distribution and geometry of different crustal constituents (Brichau et al., 2006, 2008; Ring & Layer, 2003; Ring, Brandon, et al., 1999). Thus, accurately determining the exhumation mechanism(s) responsible for unroofing a particular package of rock is critical to reconstructing the evolution of an orogen, as co‐existing exhumation styles may result in highly complex geometric configurations of local‐ and regional‐scale structural piles.

In a previous contribution, we discriminated among two structural domains within a tectonic window exposed on the island of Evia in the NW Aegean Sea (Ducharme et al., 2022). Deep structural levels of the lowermost thrust sheet (Basal Unit), composed mainly of marbles, were interpreted as preserving structures reflecting the comprehensive deformational history of the unit, whereas its uppermost structural levels, coinciding largely with a package interpreted as metamorphosed Eocene flysch, were reported to exhibit a comparatively simpler structural record. A broadly sub‐horizontal foliation in the latter domain was attributed to an extensional shear zone (Evia Shear Zone; ESZ) operating with an oblate coaxial strain component, which produced ductile and brittle‐ductile structures recording extension in the transport‐normal (i.e., Y) direction during overall top‐to‐NE general flattening. The structure was proposed to have facilitated Oligo‐Miocene unroofing of the Basal Unit via simultaneous operation as a low‐angle normal fault and ductile thinning of the shear zone (Ducharme et al., 2022).

Our new work further interrogates the premises of the ductile thinning model on southern Evia using petrofabric analysis of deformed quartz veins and paired in situ ^40^Ar/^39^Ar and ^87^Rb/^87^Sr geochronology. Stretched quartz veins are important structures that record the oblate flattening strain component within the ESZ (cf. Figure 3d of Ducharme et al., 2022), and may record, depending on the timing of their formation, distinct intervals of the progressive strain history compared to previously investigated quartzites of the region (Xypolias et al., 2003, 2010). The diverse lithological constituents of the Basal Unit flysch succession record variable finite strain intensities, attesting to a complex bulk rheology. Thus, quartz veins hosted by different lithotypes may record c‐axis distributions that do not all capture the same deformation conditions. Rheological heterogeneity may likewise influence the strain‐sensitive white mica ^40^Ar/^39^Ar and ^87^Rb/^87^Sr geochronometers, which have been shown to record different dates depending on host rock competency and composition (Barnes et al., 2023; Cossette et al., 2015; Laurent et al., 2021; Ribeiro et al., 2023). Our data show that rheologically heterogeneous parts of an orogenic section, such as intercalated diverse sedimentary units and mélanges, may localize structures that contribute significantly to exhumation, but that these structures may be obscured by an apparently incoherent finite strain record at the scale of the shear zone.

## 2 Tectonic Setting

**Figure 1:**
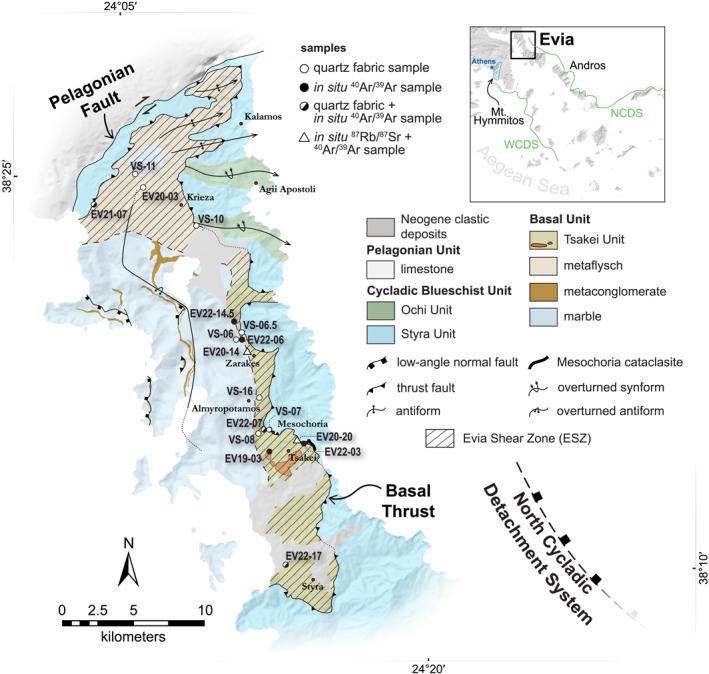
Geological map of southern Evia. Inset map shows the Cyclades with the North Cycladic Detachment System (NCDS) and West Cycladic Detachment System (WCDS) indicated. Modified after Katsikatsos (1991a, 1991b) and Ducharme et al. (2022).

Southern parts of Evia island represent the northwestern limits of the Attic‐Cycladic Crystalline Belt (ACCB), a high pressure‐low temperature (HP‐LT) metamorphic massif belonging to the greater Greek Hellenides (Figure 1). The ACCB is composed of metamorphosed equivalents to the Pindos and Gavrovo‐Tripolitza units on mainland Greece, the Peloponnese, and Crete, alongside a crystalline basement, that were subducted below (and accreted outboard of) the Pelagonian Zone along the long‐lived Hellenic subduction zone. The Cycladic Blueschist Unit (CBU) comprises a diverse assortment of marbles, clastic metasedimentary, and (dominantly mafic) metavolcanic rocks interpreted to represent subducted fragments of the Pindos Unit (e.g., Bonneau, 1984; Dürr et al., 1978; Papanikolaou, 1987; Ring et al., 2010). The CBU sustained early Eocene (c. 50 Ma) HP‐LT metamorphism prior to its unroofing since the late Eocene (e.g., Jacobshagen, 1986; Jolivet et al., 2010; Papanikolaou, 1987; Ring et al., 2010). The CBU is underlain in the Cyclades either by a crystalline basement of disputed affinity (Huet et al., 2009; Zlatkin et al., 2018), or by the Basal Unit, a Triassic‐Eocene platform carbonate succession capped by Eocene flysch that has been correlated with the Gavrovo‐Tripolitza unit (Dubois & Bignot, 1979; Katsikatsos et al., 1976; Ring et al., 2010; Schermer et al., 1990). The Basal Unit is exposed in tectonic windows across the Cyclades and in continental Greece, interpreted as representing fragments of the Gavrovo‐Tripolitza Unit that were subducted and tectonically juxtaposed below the CBU along the regional‐scale Basal Thrust (Ring et al., 2010). The Basal Unit records relict mineralogical evidence of a separate, more recent HP‐LT metamorphic episode (Ducharme et al., 2022; Gerogiannis et al., 2021; Shaked et al., 2000).

A significant body of work on the ACCB has focused on the CBU, and thus the geodynamic evolution of the Cyclades is best understood with respect to this unit. The exhumation history of the CBU is broadly subdivided into two stages (Jolivet et al., 2010; Ring et al., 2010). The earliest + increments of exhumation, commonly termed 'syn‐orogenic' exhumation, were likely accomplished via wedge extrusion during continued contraction in the Aegean (Jolivet et al., 2010; Peillod et al., 2024; Ring, Will, et al., 2007; Uunk et al., 2022). Syn‐orogenic stages of exhumation were likely responsible for the majority of total unroofing experienced by the CBU, and may have produced internal imbrication by SW‐vergent thrusting within the extruding wedge (Grasemann et al., 2018).

Later stages of exhumation, hereafter “post‐orogenic,” were dominated by bivergent extension along systems of ductile‐then‐brittle low‐angle normal faults, including the North (NCDS) and West Cycladic Detachment Systems (WCDS; Jolivet et al., 2010; Grasemann et al., 2012; Figure 1). These structures developed in response to slab rollback and incipient back‐arc formation since the early Miocene. Despite only facilitating the shallowest ∼15 km of exhumation for most of the CBU (e.g., Ring & Kumerics, 2008; Ring & Layer, 2003), the major detachment faults profoundly modified the geometry of the exhuming crust and exert a dominant control on the present day exposures of the different lithostratigraphic units via ductile strain in their footwalls and segmentation of the ACCB into crustal‐scale boudins (Jolivet et al., 2004, 2010).

### 2.1 Geology of Southern Evia

The bedrock of southern Evia is composed primarily of the CBU and Basal Unit (Figure 1). The CBU on Evia is subdivided into Styra and Ochi units, which are predominantly composed of marble and mafic metavolcanic rock, respectively (Papanikolaou, 1987; Katsikatsos, 1991a, 1991b; Shaked et al., 2000). These rocks attained peak metamorphic conditions of ∼400–460°C and ∼12 kbar, with retrograde mineral assemblages suggesting greenschist facies overprint at <350°C and <8 kbar (Ducharme et al., 2022; Katzir et al., 2000; Shaked et al., 2000). A third subdivision, the Tsakei Unit, has been applied in some interpretations to metapelitic schist underlying the Styra Unit (Katsikatsos, 1991a, 1991b), but these rocks are assigned to the Basal Unit in other interpretations (Ducharme et al., 2022; Shaked et al., 2000). The Basal Unit, first identified on Evia and locally termed the Almyropotamos Unit (Avigad & Garfunkel, 1989; Katsikatsos et al., 1976), is exposed in the Almyropotamos tectonic window, a major structural discontinuity occupying a significant portion of the island. The Almyropotamos window is, in principle, bounded by an exposure of the Basal Thrust (Ring, Glodny, et al., 2007); however, the structure lacks comprehensive description, likely because it is either poorly exposed or has been obscured by reworking of the contact during exhumation (Ducharme et al., 2022; Ring, Glodny, et al., 2007). Due to the uncertainty regarding the Tsakei Unit, the tectonostratigraphic position of the tectonic contact is also disputed: it has been interpreted to lie either consistently at the base of the Styra Unit (Ducharme et al., 2022; Shaked et al., 2000), or is otherwise placed below the Tsakei Unit at the southern limits of the tectonic window (Katsikatsos, 1991a, 1991b; Ring, Glodny, et al., 2007). Although the presence of large (up to ∼100 m diameter) metabasic and serpentinitic blocks in the Tsakei Unit is the primary justification for its discrimination from the flysch succession, both units feature hallmarks of tectonic sedimentation like marble olistoliths and locally high densities of smaller scale lithological heterogeneities (Abbate et al., 1970; Katsikatsos, 1991a; Ring, Glodny, et al., 2007).

Compared with that of the CBU, the geodynamic and metamorphic histories of the Basal Unit are poorly known. Evidence from the weakly to unmetamorphosed Gavrovo‐Tripolitza exposures in the Peloponnese and Crete indicate it entered the Hellenic subduction zone between c. 36 and 29 Ma (Sotiropoulos et al., 2003; Thomson et al., 1998). Proposed timing for peak HP metamorphism recorded by the Basal Unit, meanwhile, spans the Oligocene (Ducharme et al., 2022; Shaked et al., 2000) to early Miocene epochs (Gerogiannis et al., 2021; Ring & Layer, 2003; Ring, Glodny, et al., 2007), largely resulting from contrasting interpretations of early Miocene phengite ^87^Rb/^87^Sr and ^40^Ar/^39^Ar dates either as recording deformation or metamorphism. The lithostratigraphic interval separating the Basal Unit and Styra (CBU) marbles is the main source of samples yielding such controversial dates (Ducharme et al., 2022; Ring, Glodny, et al., 2007). Rocks within this interval are divided, in some interpretations, into Basal Unit flysch and Tsakei Unit (Katsikatsos, 1991a, 1991b), whereas others consider it as a continuous exposure of Basal Unit flysch (Figure 1; Ducharme et al., 2022; Shaked et al., 2000). Regardless of interpretation, the interval comprises pelitic, carbonate, and quartzose schists and quartzites, intercalated at the meter scale, with carbonate schists becoming volumetrically dominant toward the base of the unit. The schistose package is everywhere overlain by massive to foliated Styra Unit marbles. Symmetrical foliation‐oblique boudinaged quartz veins and conjugate brittle‐ductile faults observed perpendicular to the regional stretching lineation, along with lineation‐parallel top‐to‐NE non‐coaxial structures, led Ducharme et al. (2022) to interpret an oblate finite strain ellipsoid for the schists that corresponds to exhumation by simultaneous flattening and top‐to‐NE normal sense displacement. Due to the position of these structures, which the authors documented uniquely at structural levels coinciding with the schists, Ducharme et al. (2022) proposed that finite strain there corresponds to the so‐called Evia Shear Zone, a structure partly responsible for syn‐ to post‐orogenic exhumation of the Basal Unit on Evia.

Shaked et al. (2000), Katzir et al. (2000), and Ducharme et al. (2022) have reported detailed petrological descriptions of Basal Unit lithotypes on Evia in the context of estimating PT conditions. The Basal Unit attained maximum metamorphic conditions of ∼11 kbar and ∼400°C (Shaked et al., 2000), prior to greenschist‐facies overprint during unroofing that intersected conditions of ∼7 kbar and ∼310°C (estimate obtained from a Tsakei schist; Ducharme et al., 2022). Zircon (U‐Th)/He data show that the entire structural pile of southern Evia had exhumed into the brittle crust by the middle Miocene (c. 17–15 Ma), thus implying a rapid subduction and unroofing history relative to that of the CBU (Ducharme et al., 2022). To accommodate the short apparent interval between subduction, peak HP metamorphism, and exhumation, Ducharme et al. (2022) proposed a rapid transition from subduction to probable syn‐orogenic exhumation of the Basal Unit, followed by post‐orogenic exhumation that may have been synchronous with that of the CBU. Ductile thinning along major tectonic structures, accommodated either by pure shear (Xypolias et al., 2003, 2010) or flattening (Ducharme et al., 2022) strain in schists that divide the Basal Unit and CBU marbles, may have partially facilitated the rapid exhumation of the Basal Unit on Evia and its neighboring exposures on the Attic Peninsula.

## 3 New Field Observations

**Figure 2:**
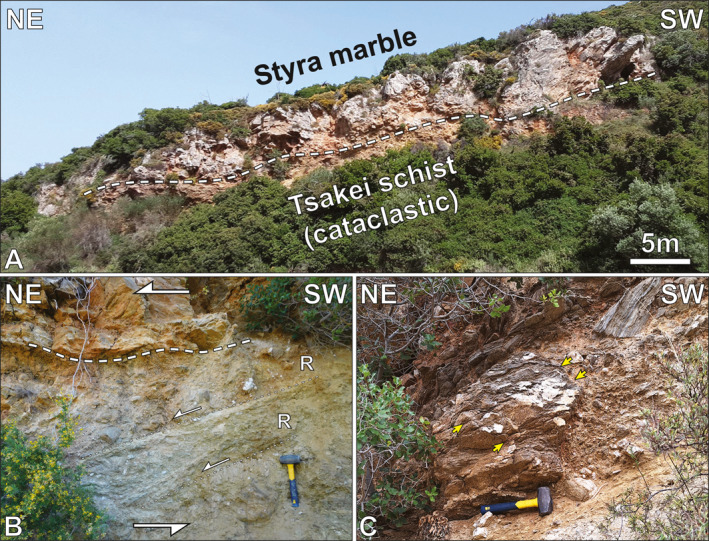
Field documentation of the Mesochoria cataclasites. (a; zone 35°N, 258568°E, 4236248°N) Cliff face exposing Styra Unit marbles directly overlying cataclasized Tsakei schists. (b; same location as a) Multi‐generational cataclasite below little deformed Styra marble. Synthetic Riedel shears (R) indicate top‐to‐NE shear sense. (c; same location as A) Schist block structurally above the cataclasite horizon showing axial planar cleavage (yellow arrows) at a high angle to foliation within the block. Scale: hammer: 27 cm long.

The contact between the Styra marbles and the underlying schists is exposed along the road connecting the village of Mesochoria and the beach of the same name (Figures 1 and 2a). Multi‐generational ultracataclasites occur below the marble, dipping ∼30° toward the NE, separated from intact Styra marbles by fragmented marble scree. Synthetic Riedel shear developed alongside striations plunging ∼20° toward NE‐ENE indicate the cataclasites accommodated top‐to‐NE brittle displacement (Figure 2b). Randomly oriented blocks of veined mafic and pelitic schist, up to a meter in diameter, are dispersed throughout the cataclasites. The upper contact with the marbles is continuous for a kilometer, in exposures along opposing cliffs and in roadcuts to the north and south.

**Figure 3:**
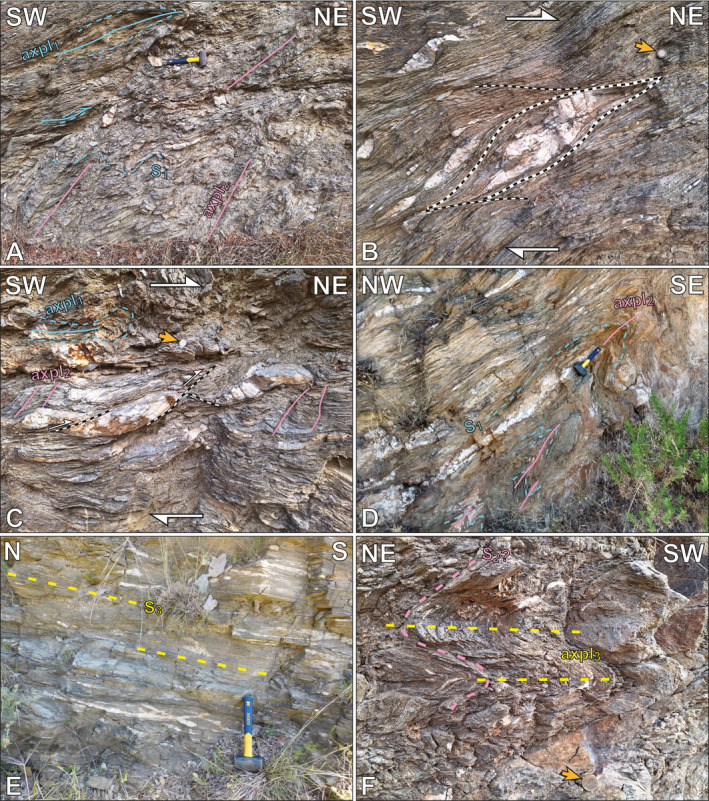
Photographs showing representative structures in the Basal Unit flysch of southern Evia. (a; zone 35°N, 257753°E, 4236238°N) A shallowly dipping foliation (S_1_; avg. strike/dip 234°/31°) shows undulations related to NW‐SE trending folds (F_2_). A W‐dipping axial plane cleavage (avg. strike/dip 180°/56°) cross‐cuts the foliation (thick dashed yellow line). Older, now rootless intrafolial isoclinal F_1_ folds (thin dotted line: fold trace; thin solid line: axial plane) subparallel (21°–265°) to the exposed face. (b; same location as A) S/C‐type shear bands (thick dotted line) showing top‐to‐NE shear sense. (c; same location as a) Tiling of imbricated quartz vein segments (thick dotted lines) indicating top‐to‐NE shear sense. The W‐dipping axial plane cleavage (thick dashed line) resembles false antithetic C′‐type shear bands. (d; zone 35°N, 257328°E, 4236737°N) Tight to isoclinal F_2_ folds of S_1_. Axial plane dips moderately NW (232°/44°) with a NW‐plunging fold axis (35°–324°). Sample location EV20‐20. (e; zone 35°N, 254766°E, 4237485°N) Representative penetrative gently E‐dipping S_3_ foliation (strike/dip 353°/25°) from sample locality EV22‐07. (f; zone 35°N, 254513°E, 4239855°N) Spaced expression of S_3_ cleavage in a carbonate‐silicate schist. Orange arrows indicate location of scale. Scales: hammer: 27 cm long; 1€ coin: 23 mm diameter.

**Figure 4:**
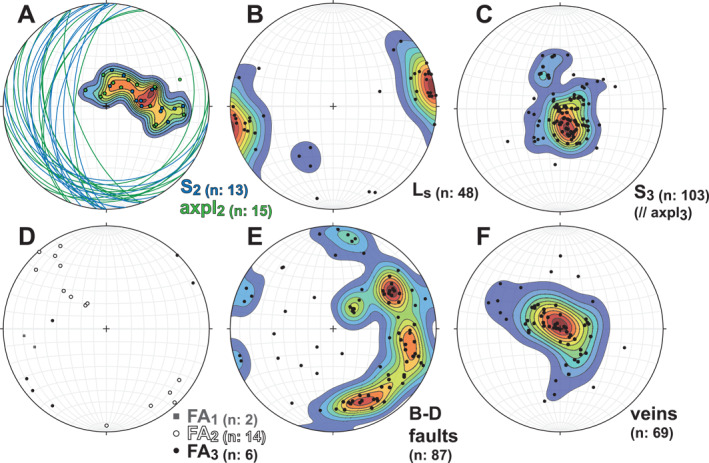
Equal area lower hemisphere stereonet plots of structural data from the Basal Unit schists of southern Evia. All planes are plotted as poles, except in (a) where both planes and poles are plotted. Contour interval is 10% of the maximum point density. Abbreviations: S—foliation; Ls—stretching lineation; axpl—axial plane cleavage; FA—fold axis; B‐D—brittle‐ductile.

Interstratified pelitic and carbonate schists structurally below the cataclasites dip gently to the NW and contain two discernible fold generations: (a) NE‐trending, rootless intrafolial isoclinal folds (F_1_) with axial planes parallel to the local main foliation (S_1_); and (b) a NW‐trending, close to isoclinal fold generation with axial planes dipping moderately or gently west (F_2_). The latter folds are associated with a prominent W‐dipping axial plane cleavage (axpl_2_) that cross‐cuts the metamorphic foliation, which itself appears axial planar to the earlier intrafolial folds (axpl_2_; Figures 3a and 4a). Blocks within the overlying cataclasite display a spaced cleavage at high angles to foliation within the blocks, resembling axpl_2_ cleavage observed in the footwall (Figure 2c). Tiling and sigmoidal quartz vein boudins alongside a NE‐SW stretching lineation, consistent with lineations documented throughout the structural pile, record top‐to‐NE shear sense structurally below the cataclasite (Figures 3b, 3c, and 4b). As the structures and related foliations recording the kinematics are not deflected or cross‐cut by the axpl_2_ cleavage, they are apparently synchronous with or post‐date the F_2_ folds. Notably, despite schist occurrences <30 m structurally below the NE‐dipping cataclasites, the schists themselves dip NW, marking an abrupt ∼90° change in dip direction.

**Figure 5:**
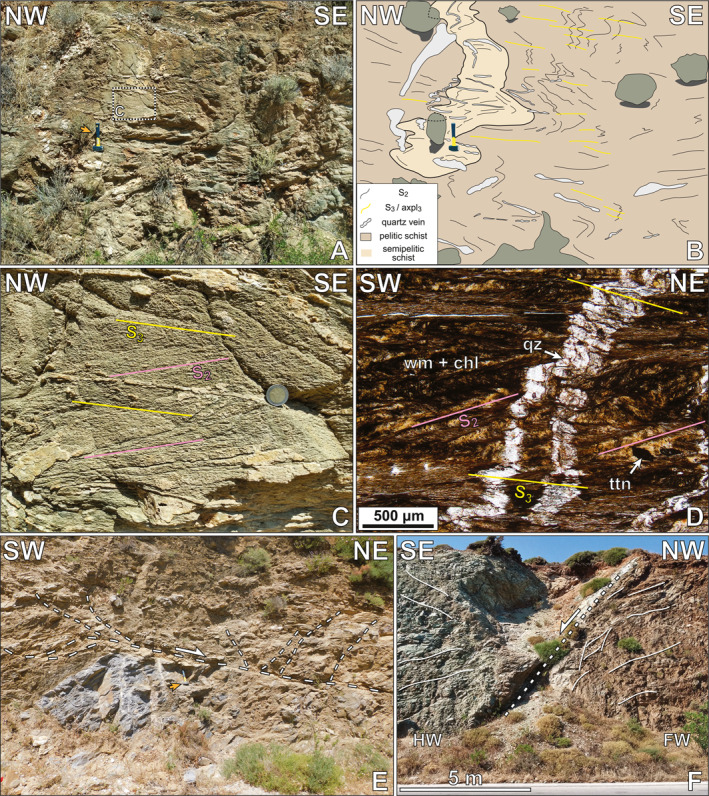
Field photographs and micrographs showing strain partitioning among juxtaposed lithotypes in the Evia Shear Zone. (a–d; zone 35°N, 253314°E, 4245312°N) Photograph (a, c), interpretive sketch (b) and micrograph (d) of a competent subvertical semipelitic schist deformed within less competent chlorite schists. (a, b) The main outcrop structure shows a gently E‐dipping spaced crenulation cleavage (S_3_) that is axial planar to the dominant folds (axpl_3_). (c) The competent lens preserves a shallowly W‐dipping cleavage (S_2_) between a younger fabric and quartz veins parallel to S_2_. (d) Micrograph showing preservation of W‐ dipping S_2_ cleavage within microlithons. Note quartz vein, which shows folding only about axial planes parallel to S_3_ cleavage. Thin section cut parallel to NE‐trending L_S_, oblique to the outcrop face. (e; zone 35°N, 254659°E, 4237460°N) Low‐angle brittle‐ductile normal fault and associated high‐angle conjugate normal faults localized along the contact between pelitic schist and a marble block. (f; zone 35°N, 254910°E, 4236253°N) High‐angle, SE‐dipping normal fault dividing a metabasic block (HW) from Tsakei schists (FW). Mineral abbreviations after Whitney and Evans (2010). Orange arrow indicates location of scale. Scale: hammer: 27 cm long; 2€ coin: 26 mm diameter.

**Figure 6:**
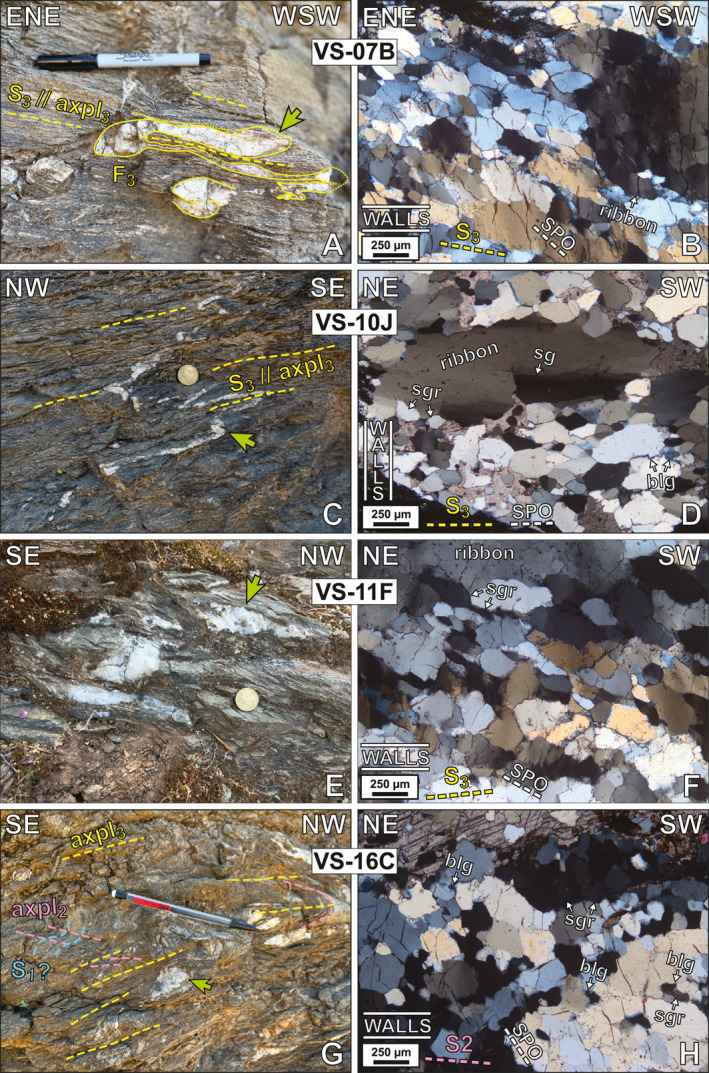
Field photographs and micrographs of quartz veins used for petrofabric analysis. Lines labeled 'walls' on micrographs indicate host rock‐vein orientation relative to photo. Yellow dashed lines indicate the interpretation of foliation generation and its orientation relative to the micrograph, and white dashed lines indicate orientation of the quartz shape preferred orientation (SPO). (a, b; zone 35°N, 254783°E, 4237525°N) Vein sample VS‐07B is isoclinally folded in outcrop with the gently W‐dipping macroscopic host foliation parallel to the axial plane. Recrystallized quartz within the vein shows an oblique SPO indicating top‐to‐ENE shear sense. Note similar sizes of subgrains in the rightmost quartz ribbon and surrounding finer grains. (c, d; zone 35°N, 250655°E, 4252375°N) Isoclinally folded vein sample VS‐10J shows alternating pinch‐ and‐swell of foliation‐parallel limbs and isoclinal folding of foliation‐normal hinges. Connecting segments of the vein are missing, perhaps due to pressure solution. Quartz shows an SPO parallel to the host foliation. Smaller grains show well‐defined grain boundary triple junctions. (e, f; zone 35°N, 245404°E, 4256238°N) Sample VS‐11F occurs as several disconnected segments. Quartz exhibits low‐amplitude bulging at grain boundaries and an oblique SPO consistent with top‐to‐NE shear sense. (g, h; zone 35°N, 254538°E, 4239847°N) Vein sample VS‐16C is an isolated pocket of quartz occupying the hinge of a NE‐SW trending fold in an outcrop preserving two axial plane cleavages. Quartz shows bulging and sutured grain boundaries. Green arrows indicate the approximate area shown in thin section. Abbreviations: blg: grain boundary bulging; ribbon: monocrystalline quartz ribbon; sg: subgraining; sgr: subgrain rotation. Scales sizes: pen in (a): 15 cm; 50¢ coin in (c, e): 24 mm diameter; pen in (g): 14.5 cm.

Exposures of upper structural levels of the flysch maintain a structure dominated by m‐scale, E‐vergent folding of a pre‐existing, now W‐ to NW‐dipping tectonic fabric (Figure 3d). Deeper structural levels, approaching the lower contact with the Basal Unit marbles, more commonly show dominant sub‐horizontal foliations that define either a tightly spaced (<1 cm) crenulation cleavage or a penetrative foliation (Figures 3e and 4c). More carbonate‐ or quartz‐rich lithotypes intercalated with schists commonly retain evidence of earlier tectonic fabrics, preserved in folds or microlithons associated with comparatively widely spaced expressions of the same sub‐horizontal cleavage, dissecting an older cleavage and related close to isoclinal folding of an older foliation (Figures 3, 5, and 5b). Microlithons between cleavage domains locally preserve a shallower W‐dipping cleavage truncated by the spaced sub‐horizontal cleavage (Figure 5c). Quartz veins cross‐cutting the dominant outcrop‐scale foliation at high angles show no evidence of deformation by the W‐dipping cleavage but are folded about the sub‐horizontal foliation (Figure 5d). In outcrops apparently showing a single penetrative foliation, the folded quartz veins possess axial planes consistently parallel to that foliation (Figures 6a and 6c). Due to the cross‐cutting relationship observed between the two cleavages and the observation that quartz veins are deformed by only one of the two, we infer that the variably spaced sub‐horizontal cleavage is a third axial planar cleavage (axpl_3_), related to NE‐trending, flat‐lying, close to isoclinal F_3_ folds that refold and/or disjunctively crenulate axpl_1_. When closely spaced, as frequently documented at moderate to deep structural levels of the flysch, the F_3_ axial plane cleavage defines the dominant penetrative foliation (S_3_).

More discrete structures, such as shear bands and faults, localize preferentially in pelitic lithotypes along contacts with interspersed blocks of marble, quartzite, or metabasite (Figure 5e). A prominent example is the localization of normal faults along the metabasic blocks within the Tsakei Unit along the highway west of Raptei (Figures 1 and 5f).

## 4 Analytical Methods

### 4.1 Quartz c‐Axis Fabric Analysis

Twelve samples containing one or more quartz veins were selected for quartz petrofabric analysis. Oriented standard 30 μm polished thin sections of the samples were scanned with a Russell‐Head Instruments G60+ automated fabric analyzer at the University of British Columbia, Okanagan (Kelowna, Canada). Analyzed sections are photographed from nine different angles using a range of polarizer and accessory plate orientations (Larson, 2018). The resulting images are processed together to develop a statistical rendering of the orientation of quartz c‐axes, an achsenverteilungsanalyse diagram (Sander, 1950), with minimal detectable deviation from other c‐axis determination techniques (Peternell et al., 2010; Wilson et al., 2007). Quartz c‐axis orientations are stored at the pixel scale in the captured images, and c‐axis orientations (one per quartz grain) are manually selected in the Investigator companion software. Between 609 and 5,735 individual grain c‐axis orientations were selected per sample from among quartz hosted in the veins. All distinguishable grains yielding a well‐resolved c‐axis orientation were included in the data set, proceeding unidirectionally through a scan to minimize bias in the c‐axis populations.

Selected c‐axis orientations were processed using a custom script written in R (Larson, 2023). The script handles statistical analysis of the c‐axis orientation data, including definitions of kernel widths and number of bins. These preferences are then used to generate scatter and contour plots on equal‐area lower hemisphere stereonet projections. The script also calculates fabric strength parameters including multiples of uniform density (m.u.d.), the pole figure index J_pf_ of Mainprice et al. (2015), and fabric cylindricity parameters P, G, R, and B after Vollmer (1990).

### 4.2 In Situ ^40^Ar/^39^Ar Geochronology

A suite of standard 30 μm polished thin sections were prepared to examine the microstructures developed in mica from 20 samples. From among these, 13 150 μm polished thick sections were prepared with cyanoacrylate (i.e., super glue) from nine samples for in situ laser ablation ^40^Ar/^39^Ar geochronology. Thick sections were prepared using the same section of rock the thin sections were prepared from to maximize homology of visible microstructures.

Sections were thoroughly characterized and photographed using a combination of conventional transmitted and reflected light microscopy. Additional imaging was performed using a JEOL 6610LV scanning electron microscope (SEM) at the University of Ottawa to obtain backscatter electron (BSE) images, equipped with an energy dispersive spectrometry (EDS) detector for semi‐quantitative mineral identification. Once regions containing a sufficient density of inclusion‐free white mica were identified, regions of interest in the polished thick sections were isolated using a finishing saw and removed from their glass mount by dissolving the adhesive in acetone overnight. Isolated rock chips were then packaged into aluminum discs for irradiation.

Complementarily, white mica chemical compositions were determined from the counterpart thin sections via electron microprobe analysis at the University of Ottawa using a JEOL 8230 SuperProbe. Major and minor elements were determined quantitatively using a 10 μm spot size. Spot selection prioritized white mica in the same microstructural contexts as were identified as targets for in situ ^40^Ar/^39^Ar analysis, as well as white mica defining the dominant metamorphic foliation. Detailed analytical parameters may be found in Supporting Information S1.

Following a 60‐day irradiation, sample chips were mounted using a ceramic adhesive (PELCO®) on a quartz slide placed in a stainless‐steel chamber with a sapphire viewport attached to the stainless steel high vacuum extraction system and allowed to stabilize prior to analysis at the University of Manitoba (Winnipeg, Canada). Analyses were performed using a multi‐collector Thermo Fisher Scientific ARGUS VI mass spectrometer connected to a Photon Machines (55W) Fusions 10.6 CO_2_ laser to ablate target regions 200 μm × 50 μm in size.

### 4.3 In Situ ^87^Rb/^87^Sr Geochronology

Two 150 μm thick polished sections were prepared for in situ ^87^Rb/^87^Sr geochronology using an Agilent 8,900 triple‐quadrupole inductively coupled plasma mass spectrometer equipped with a reaction cell (Hogmalm et al., 2017; Zack & Hogmalm, 2016) paired to an ESL 193 Excimer laser with a TwoVol3 ablation cell in the Fipke Laboratory for Trace Element Research at University of British Columbia, Okanagan (Kelowna, Canada). The analyses followed the basic procedures outlined in Larson et al. (2023) with white mica ablated using a 50 μm diameter spot, a repetition rate of 10 Hz and a laser fluence of 4 J/cm^2^. Analyses of secondary reference materials, including the in‐house white mica MA1 (c. 350 Ma, A. Camacho, unpubl. data) and the nano‐powdered biotite Mica‐Fe (310 ± 10 Ma, Govindaraju, 1979; 305.4 ± 2.0 Ma, Rösel and Zack, 2022), yielded dates that overlap within error of those expected (347 ± 3 Ma and 306 ± 3 Ma, respectively). Isochron calculations excluded analyses with high uncertainty, using a >30% (2 standard error of the mean) cut‐off that resulted in the exclusion of one analysis per sample. Data processing and visualization were performed using the online version of the IsoPlotR package (Vermeesch, 2018).

## 5 Quartz Petrofabric Analysis

### 5.1 Sample Descriptions

**Figure 7:**
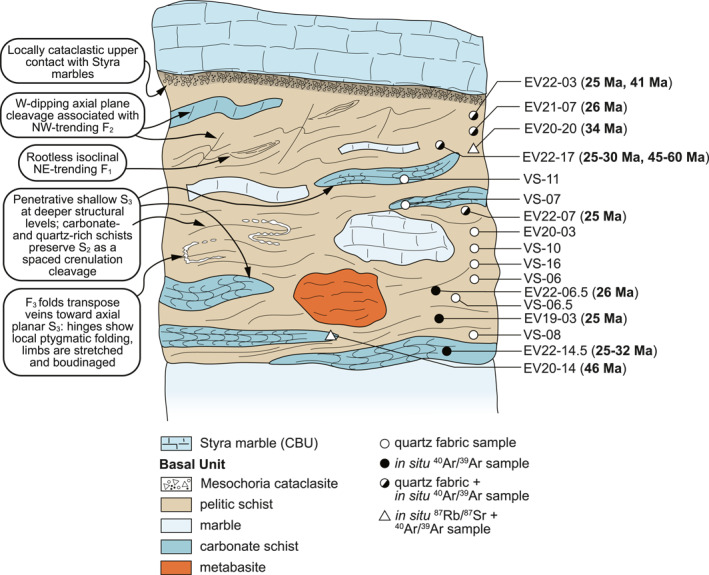
Schematic structural section of the schists on Evia showing approximate structural positions for each sample. Text bubbles on the left summarize major structural observations described in Section 3. The section considers the Tsakei Unit and Basal Unit flysch as a single continuous unit.

Veins exhibiting macroscopic evidence of deformation (i.e., pinch‐and‐swell or folding) were sampled from pelitic and carbonate schists of the Basal Unit metaflysch and Tsakei Unit to assess the presence of a crystallographic preferred orientation (CPO) in quartz (Figures 1 and 7; Figure S1 in Supporting Information S1). Veins recording deformation hosted in quartz‐rich lithotypes were comparatively rare, and thus these are unrepresented in our data. Selected samples prioritized veins containing proportionally greater quartz than carbonate material, and which displayed microstructures consistent with dynamic recrystallization of quartz (i.e., subgrain formation, sutured or bulging grain boundaries).

Most quartz veins in the study area are oriented parallel or slightly oblique to the gently dipping foliation (Figures 4f and 6). Veins displaying this relationship were either part of a continuous, laterally extensive pinch‐and‐swell quartz vein, or formed isolated sigmoids (Figure 6e). Two of the veins (VS‐07B, VS‐10J) are isoclinally folded about axial planes parallel to the penetrative foliation of their host rock (Figures 6a and 6c). Hinge sections of these veins cross‐cut foliation at a high angle and may be ptygmatic or thickened (Figures 6c and 6g), whereas their limbs are foliation parallel or oblique and elongated parallel to foliation, like the isolated vein segments described above.

Vein fill consists of variable proportions of quartz and calcite, with some veins composed solely of quartz. Veins selected for quartz c‐axis fabric analysis were dominantly coarse grained (average grain diameter >150 μm), frequently with a finer population (∼40–250 μm) surrounding coarser (500–1,000 μm) quartz ribbon porphyroclasts with abundant internal subgrains (Figure 6d). Ribbon porphyroclasts were observed in half the samples, whereas the remaining six (VS‐08H, EV20‐03, EV21‐07, EV22‐03, EV22‐07, and EV22‐17) contain comparatively equigranular polygonal aggregates exhibiting an SPO and local, slightly serrated grain boundaries, with some microstructures dominated by pinning of quartz by mica (e.g., EV20‐03, EV22‐03). Aspect ratios of the ribbon grains vary considerably, with some exhibiting nearly equant dimensions and others approaching aspect ratios of 10:1. Long axes of elongate subgrains are universally parallel to the long axis of their host ribbon. Quartz grains mantling the ribbons exhibit low‐amplitude grain boundary bulging and sutured grain boundaries, and commonly display a shape preferred orientation (SPO), either with long axes approximately normal (Figures 6d and 6h) or oblique to the vein walls (Figures 6b and 6f). Oblique SPOs indicate a consistent top‐to‐NE or ‐ENE shear sense. Deformation lamellae of sub‐basal type (with lamellae oriented between 70° and 90° from the grain c‐axis; e.g., White, 1973) and prismatic subgrains are commonly observed in both coarse older grains and finer new grains, defining a patchy undulatory extinction, although the latter are most prominently developed in relict grains. Quartz surrounding relict grains are of approximately the same size as the subgrains therein, typically between 20 and 150 μm. Some, though not all, samples show an abundance of well‐developed quartz triple junctions (Figure 6d). Additional microstructural documentation of the analyzed veins may be found in Figure S2 in Supporting Information S1.

### 5.2 Results

**Figure 8:**
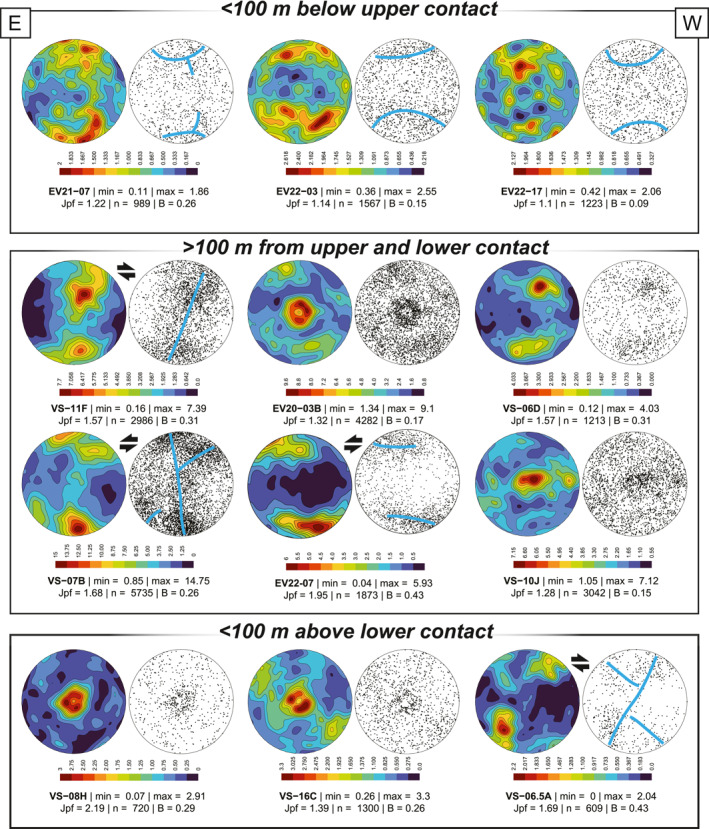
Lower hemisphere equal area projections showing contour (left) and scatter (right) plots of quartz c‐axis orientations. Shear sense interpretations are included where applicable. All plots are oriented with the foliation (XY) plane perpendicular to the stereoplot and aligned E‐W, and the stretching lineation oriented parallel to the E‐W direction (real orientations range between ENE‐WSW and ESE‐WNW). Samples are grouped according to structural position (see Figure 7).

Pole figures obtained from quartz veins deformed within the ESZ exhibit diverse c‐axis distributions (Figure 8). Most pole figures display a prominent c‐axis maximum or maxima close to Z (EV21‐07; EV22‐03; EV22‐17; VS‐07B; EV22‐07) or Y (EV20‐03B; VS‐10J; VS‐16C; VS‐08H). Remaining samples possess two maxima of subequal c‐axis concentrations along a great circle as part of a single girdle (VS‐11F) or separated by ∼30° along a great circle (VS‐06D). Finally, one sample (VS‐06.5A) shows a strong c‐axis maximum ∼30° from Y, defining the lower central segment of a girdle.

Four pole figures define distributions characterized by a small circle distribution centered on Z (EV21‐07; EV22‐03; EV22‐07; EV22‐17). The small circle segments of one such sample (EV21‐07) are connected by an off‐center girdle. Vein samples recording this type of distribution are hosted in mica schists that contain porphyroblasts of albite, and in one example epidote (EV21‐07). Carbonate constitutes a variable proportion of the veins, but is minor or absent in the pelitic host rock. With the exception of EV22‐07, which is situated deeper in the structural pile, samples defining Z‐centered fabrics were collected at relatively high structural levels of the flysch or Tsakei units, near or immediately below the upper contact with the Styra marbles (Figures 1 and 6).

Three other samples (VS‐06.5A, VS‐07B, VS‐11F), obtained largely from intermediate or lower structural levels of the Tsakei schists, yield pole figures defining asymmetric, type‐I cross‐girdles (Lister, 1977). Girdle asymmetry, resolved relative to the oriented thin sections, indicate top‐to‐WSW shear sense.

Other distributions are dominated by a single *Y*‐axis maximum, with (VS‐10J; VS‐16C) or without (EV20‐03B; VS‐08H) the additional dispersion of c‐axes along an adjoining small circle. Sample VS‐16C displays a possible inclined, asymmetric type‐II cross girdle (Lister, 1977) with one weaker limb. Finally, one sample (VS‐06D) exhibits two subequal maxima symmetrically distributed about Y and oblique to both Z and X directions.

Fabric strength, as defined using the pole figure index J_pf_ (Mainprice et al., 2015) and cylindricity (B; Vollmer, 1990) parameters, generally increases down section. The strength parameters are broadly accompanied by increases in the maximum of the m.u.d. within a given pole figure. Vein samples from high structural levels yield J_pf_ values not significantly higher than 1, and B values approaching 0, indicating a weakly defined fabric with maximum m.u.d. of ∼2. Below the structurally highest levels of the flysch, quartz c‐axis distributions delineate more well‐defined fabrics (J_pf_ > 1, B approaching 1).

## 6 Geochronology

### 6.1 In Situ ^40^Ar/^39^Ar Geochronology

Nine samples from throughout the structural thickness of the Tsakei and flysch units were selected for in situ ^40^Ar/^39^Ar geochronology (Figure 7). Three among these (EV19‐03, EV20‐14, EV20‐20) were previously dated using the multiple single‐grain total fusion method (Ducharme et al., 2022). Sample selection prioritized a variety of lithotypes dispersed throughout the purported width of the ESZ, with secondary preference applied to samples where mica‐bearing microlithons exhibit distinct microstructures (e.g., shear bands, crenulation cleavage) to assess the correlation of microstructure with measured ^40^Ar/^39^Ar dates. White mica compositions were determined via EMPA on thin sections prepared from the same sample block as the dated thick sections, targeting white mica defining both metamorphic foliation and microstructures identified during initial sample characterization. Complete analytical results for the EMPA white mica chemistry and ^40^Ar/^39^Ar geochronology are presented in Tables S1 and S2, respectively.

The dated samples are divided into four petrographically distinct groups. The first consists of albite‐mica schists, where albite is the sole porphyroblast phase (EV22‐03, EV20‐20, EV22‐17). White mica in these samples defines foliation, or more rarely occurs as fans at high angles to foliation. Moderate sized (∼50–100 μm) crystals of white mica are intimately associated with disseminated, <10 μm diameter minerals, likely clays and hematite as evinced by EMPA analyses (see below), and rarer pale green to colorless chlorite and titanite. This group of samples features numerous foliation‐parallel quartz (± carbonate) veins. Albite porphyroblasts adjacent to veins often show incipient replacement by coarse white mica crystals that do not exhibit a preferred orientation. Additionally, this group of samples exhibits variable structure: EV22‐03 contains foliation‐oblique domains of fractured and fragmented albite porphyroblasts; and EV22‐17 contains <0.5 cm thick shear bands defined by white mica and chlorite oriented at a high angle to the dominant foliation.

The second group consists of four samples that contain one or more additional porphyroblast phases as well as albite (EV21‐07, EV22‐07, EV19‐03, EV22‐06B), which are listed hereafter in order of increasing abundance. Sample EV21‐07 comprises epidote in proportion to albite as well as minor tourmaline and titanite. White mica in this sample is locally intergrown with discrete patches of green chlorite, and the phyllosilicate assemblage is highly discontinuous due to dense overgrowth by apparently post‐kinematic porphyroblasts. Sample EV22‐07 contains only tourmaline and minor titanite, and notably has ∼20% paragonite intergrown with dominant phengite and subordinate chlorite. A detailed petrographic description of sample EV19‐03 is presented in Ducharme et al. (2022), but in brief consists essentially of white mica and albite with subordinate pumpellyite and very minor foliation‐parallel brown amphibole porphyroblasts. Relics of sodic amphibole and lawsonite are preserved within albite porphyroblasts. Lastly, EV22‐06B consists of abundant <250 μm diameter porphyroblasts of epidote, along with comparatively fewer, but coarser (250–500 μm) tourmaline and titanite, all of which may be either oblique or parallel to foliation. This sample contains abundant, foliation‐parallel, thin laths of hematite intergrown with foliation‐defining white mica and minor chlorite. Sample EV22‐06B also contains infrequent mm‐scale horizons dominated by carbonate minerals, and these appear commonly as rounded inclusions in albite porphyroblasts. All samples within this group contain coarser white mica than samples of the previous group (consistently >100 μm, with crystals locally up to 250 μm), including widespread inclusion‐free domains within the foliation, well exceeding the ablation raster size. Additionally, whereas white mica predominantly defines foliation in these rocks, fan‐like phyllosilicate clusters at a high angle to foliation are present as described for the previous group, although here they are likely to comprise monomineralic chlorite or white mica. Porphyroblasts within this group overwhelmingly exhibit microstructures consistent with post‐kinematic growth, though mica locally wraps around certain porphyroblasts suggesting some syn‐kinematic crystallization.

The final two groups comprise one sample each, both containing considerable modal proportions of carbonate minerals. Sample EV22‐14.5 is a silicate‐carbonate schist defined by alternating, several mm‐scale bands of pure carbonate and bands of intergrown white mica and chlorite alongside albite porphyroblasts and minor quartz. Phyllosilicate‐rich layers exhibit a spaced crenulation cleavage with locally preserved isoclinal fold closures, as well as minor late blocky white mica and chlorite overgrowing the crenulation cleavage at a high angle. Minor carbonate minerals are also present in these layers, often as inclusions in albite porphyroblasts. Sample EV20‐14 is a schistose impure marble dominated by pure carbonate with intervening, mm‐scale horizons of white mica and chlorite with accessory apatite, constituting ∼15% of the rock volume. The phyllosilicates define a single foliation alongside the shape‐preferred orientation of carbonate minerals in adjacent domains. Like the second group, mica in both rocks occurs as apparently pure clusters well above the ablation raster size, although mica within the clusters is noticeably finer‐grained (∼50–75 μm), and several domains show sub‐micron scale intergrowth between white mica and chlorite.

In addition to recrystallized, foliation‐concordant white mica, microstructures we targeted in the dated samples include: (a) coarser‐grained, lens‐shaped white mica 'fans' defining high or moderate angles to the foliation; (b) crenulated foliations with a spaced crenulation cleavage; (c) shear bands oriented at a high angle to the adjacent foliation; (d) isolated mono‐ or polycrystalline single grains appearing within veins and/or replacing or entrained within other mineral phases; and (e) mica occupying strain shadows to porphyroblasts of albite, epidote, or tourmaline (Table 1).

**Table 1:** Summary of New In Situ ^40^Ar/^39^Ar Geochronology

Sample	Analysis	Microstructure	^40^Ar/^39^Ar date (Ma)	±1*σ* (Ma)	%^40^Ar	Mica Composition	
EV22‐03: albite‐white mica schist (*n*: 12/14)						Si (apfu)	X_Mg_
Chip 1	1	Foliation	46.8	0.9	86.0	3.36–3.42 (mean: 3.39)	0.74–0.81 (mean: 0.79)
	2	Foliation	36.8	1.0	74.6		
	3	Strain shadow	42.4	0.9	79.1		
	*4*	–	*27.3*	*1.4*	*50.0*		
Chip 2	1	Coarse, blocky, vein fill	22.9	0.9	65.8		
	2	Coarse, after albite	24.3	1.0	73.6		
	3	Coarse, blocky, vein fill	23.9	0.8	77.6		
	4	Strain shadow	35.2	1.1	74.3		
Chip 3	1	Strain shadow	37.8	0.9	72.4		
	*2*	–	*22.5*	*1.2*	*46.9*		
	3	Coarse, after albite	28.2	0.8	71.9		
	4	Foliation	44.7	0.7	82.0		
	5	Foliation	38.1	0.8	79.3		
	6	Coarse, high‐angle fan	25.1	0.7	82.8		
EV21‐07: epidote‐white mica schist (*n*: 8/8)						Si (apfu)	X_Mg_
Chip 2	1	Foliation	27.0	0.9	84.7	3.12–3.52 (mean: 3.36)	0.58–0.76 (mean: 0.69)
	2	Foliation	23.7	0.9	81.5		
	3	Fan	28.5	0.9	81.7		
	4	Foliation	26.6	0.9	85.9		
Chip 3	1	Foliation	27.1	0.7	73.9		
	2	Foliation	23.4	0.7	80.7		
	3*	Strain shadow	36.6	0.8	85.0		
	4	Strain shadow	27.0	0.7	85.1		
EV20‐20: albite‐white mica schist (*n*: 8/8)						Si (apfu)	X_Mg_
Chip 1	1	Foliation	34.9	1.3	81.7	3.35–3.46 (mean: 3.41)	0.67–0.73 (mean: 0.70)
Chip 2	2	Foliation	29.8	2.2	63.2		
	3	Fan	34.4	1.2	82.6		
	4	Foliation	31.3	0.8	59.2		
Chip 4	1	Foliation	35.9	1.0	93.6		
	2	Foliation	29.3	1.6	69.9		
	3	Fan	40.6	1.4	78.9		
	4	Foliation/strain shadow	32.4	1.4	85.9		
EV22‐17: albite‐white mica schist (*n*: 12/12)						Si (apfu)	X_Mg_
Chip 1	1	Foliation	34.0	1.4	63.8	3.33–3.59 (mean: 3.46)	0.72–0.82 (mean: 0.77)
Chip 2	1	Coarse, blocky, vein fill	30.4	0.5	77.0		
	2	Coarse, blocky, vein fill	24.5	1.2	55.2		
	3	Coarse, blocky, vein fill	27.4	1.1	56.6		
	4	Coarse, blocky, vein fill	30.1	0.6	74.9		
Chip 3	1	Shear band	29.4	0.7	84.3		
	2	Shear band	40.0	0.6	89.6		
	3*	Foliation	61.2	3.9	58.2		
	4	Shear band	46.5	0.7	86.9		
Chip 4	1	Shear band	39.8	0.5	88.1		
	2*	Shear band	57.2	0.4	90.5		
	3	Foliation	35.2	0.9	50.9		
EV22‐07: paragonite‐tourmaline schist (*n*: 11/13)						Si (apfu)	X_Mg_
Chip 2	1^pg^	Foliation	31.8	4.4	87.8	phengite: 3.24–3.39 (mean: 3.32)	phengite: 0.60–0.66 (mean: 0.65)
	2^pg^	Foliation	26.4	1.8	66.0		
	*3* ^*pg*^	–	*25.8*	*1.5*	*40.2*		
	4	Foliation	21.1	0.8	72.3		
	5^pg^	Fan	42.0	0.7	71.6		
	6*	Foliation	55.3	0.6	81.0		
Chip 4	1^pg^	Foliation	28.6	2.6	71.6	paragonite: 2.96–3.02	paragonite: N/A
	2	Fan	39.5	0.6	89.2		
	3^pg^	Foliation	29.4	2.7	82.6		
	4	Fan	35.2	0.7	77.4		
	*5* ^*pg*^	–	*29.5*	*3.5*	*33.7*		
	6	Foliation	22.5	0.8	75.4		
	7	Foliation	23.7	0.7	82.0		
EV19‐03: pumpellyite‐albite schist (*n*: 11/11)						Si (apfu)	X_Mg_
Chip 1	1	Foliation	25.0	1.4	83.3	3.38–3.61 (mean: 3.45)	0.84–0.88 (mean: 0.85)
	2	Foliation	25.7	1.0	88.5		
	3	Foliation	24.7	1.1	83.8		
	4	Foliation	23.3	1.0	74.4		
	5	Foliation	26.5	0.9	89.8		
	6	Foliation	26.5	0.9	90.3		
Chip 2	1	Fan, high‐angle	22.9	1.1	85.9		
	2	Fan, high‐angle	25.3	1.2	88.2		
	3	Fan, high‐angle	23.3	1.5	90.5		
	5	Fan, high‐angle	21.7	1.2	79.7		
	6	Fan, low‐angle, large single crystal	25.1	1.2	88.9		
EV20‐14: schistose impure marble (*n*: 6/9)						Si (apfu)	X_Mg_
Chip 1	1	Foliation, micaceous microlithon	43.9	1.0	88.0	3.50–3.64 (mean: 3.54)	0.73–0.79 (mean: 0.76)
	2	Foliation, micaceous microlithon	37.6	1.1	49.4		
	3	Foliation, micaceous microlithon	38.6	1.0	57.1		
	4	Foliation, micaceous microlithon	41.6	0.9	52.2		
	5	Foliation, micaceous microlithon	42.0	1.1	58.3		
	*6*	*Foliation, micaceous microlithon*	*40.6*	*1.4*	*38.8*		
Chip 2	*1*	*Foliation, micaceous microlithon*	*47.0*	*1.4*	*48.1*		
	*2*	*Foliation, micaceous microlithon*	*49.4*	*1.6*	*44.7*		
	3	Foliation, micaceous microlithon	45.8	1.0	79.8		
EV22‐06B: epidote‐albite‐tourmaline schist (*n*: 8/8)						Si (apfu)	X_Mg_
Chip 1	1	Fan, high‐angle	22.8	0.6	79.5	3.30–3.47 (mean: 3.37)	0.54–0.65 (mean: 0.60)
	3	Strain shadow	29.1	0.5	102.0		
	4	Shear band	25.7	0.5	89.6		
Chip 2	1	Strain shadow	23.0	0.6	98.4		
	2	Strain shadow	30.3	0.6	93.4		
	3	Foliation	25.4	0.7	60.1		
	4	Foliation	23.3	0.6	83.0		
	5	Foliation	25.9	0.6	71.7		
EV22‐14.5: carbonate‐silicate schist (*n*: 12/12)						Si (apfu)	X_Mg_
Chip 1	1	Foliation‐parallel cleavage domain	27.5	0.6	89.0	3.48–3.64 (mean: 3.57)	0.76–0.81 (mean: 0.79)
	2	Crenulated relic foliation	31.4	0.6	83.6		
Chip 2	1	Foliation‐parallel cleavage domain	28.1	0.6	88.7		
	2	Crenulated relic foliation	26.7	0.5	80.2		
	3	Isolated blocky crystal in chlorite	25.0	0.8	75.0		
	4	Foliation	29.5	0.7	93.3		
	5	High‐angle coarse blocky crystals	25.9	0.6	77.8		
	6	Foliation‐parallel cleavage domain	30.3	0.6	87.4		
	7	Crenulated relic foliation	31.6	1.0	83.2		
Chip 3	1	Foliation‐parallel cleavage domain	31.5	0.7	62.3		
	2	Crenulated relic foliation	31.4	0.6	69.6		
	3	Foliation‐parallel cleavage domain	31.8	0.7	76.4		

*Note*. Italics indicate analyses rejected due to low ^40^Ar*. *Analysis excluded from weighted mean calculation in Figure 10. pg—paragonite.

**Figure 9:**
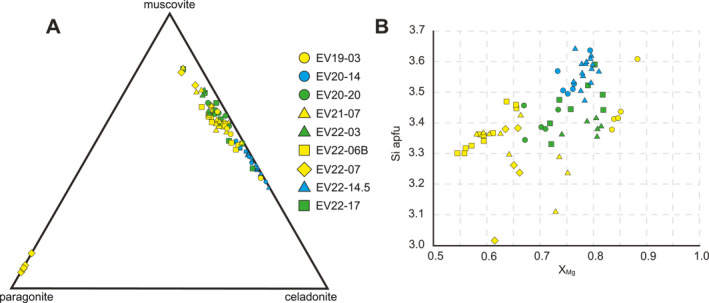
Compositional diagrams for white mica in samples selected for in situ ^40^Ar/^39^Ar and ^87^Rb/^87^Sr geochronology. (a) White mica ternary plot. (b) White mica Si apfu versus X_Mg_ plot. Symbols are grouped by lithotype: blue—silicate‐carbonate schist and schistose impure marble; green—albite‐mica schists; yellow—pelitic lithotypes containing porphyroblasts of albite and one or more of tourmaline, epidote, or pumpellyite.

Potassic white mica is overwhelmingly phengite, with limited spot analyses indicating muscovitic compositions with Si < 3.2 apfu (Figure 9). Mica chemistry within a sample does not vary systematically with microstructure. Carbonate‐rich lithotypes contain mica with the highest Al‐celadonite component, whereas phengite in pelitic samples contained overall higher Fe^2+^. However, pelitic schist sample EV19‐03 contained more magnesian phengite than even the carbonate‐rich samples. Despite providing stoichiometrically reasonable microprobe data, most analyses from the first petrographic group (EV20‐20, EV22‐03, EV22‐17) failed the recalculation exclusion criteria outlined by Vidal and Parra (2000), perhaps implying sub‐microscopic intergrowths or inclusions of a chemically similar mineral (e.g., illite).

**Figure 10:**
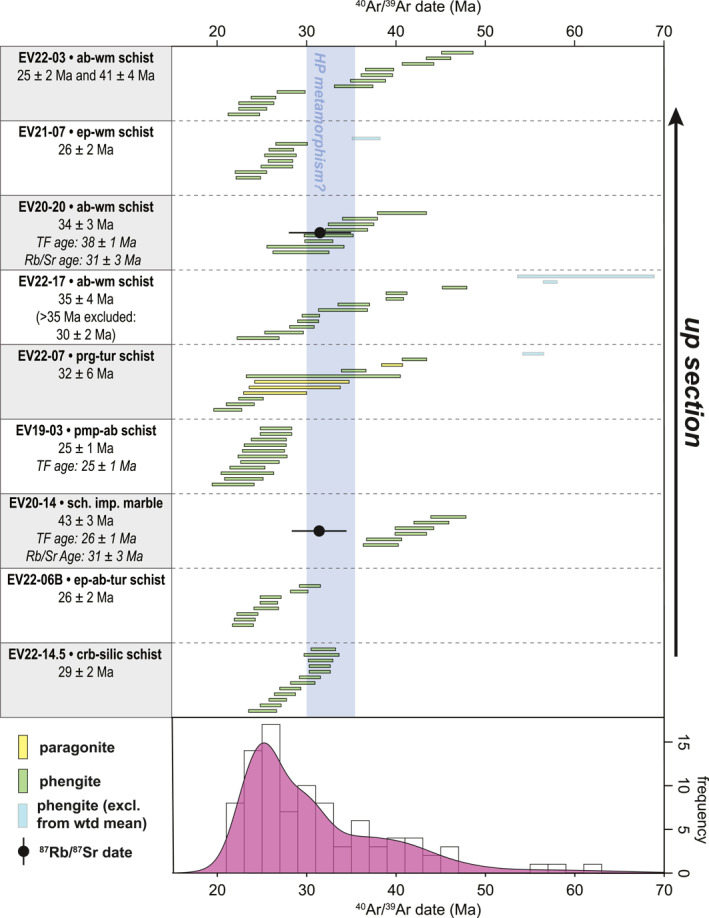
Summary of new in situ white mica ^40^Ar/^39^Ar geochronology from the Basal Unit and Tsakei schists of southern Evia. Samples are ordered from structurally lowest (bottom) to structurally highest (top). Bars indicate 1σ errors. TF age: single‐grain total fusion age (Ducharme et al., 2022). Blue bar indicates possible window of peak HP‐LT metamorphism for the Basal Unit based on data from the Gavrovo‐Tripolitza Zone (Sotiropoulos et al., 2003; Thomson et al., 1999). Refer to Table 1 for unabbreviated lithotype names.

**Figure 11:**
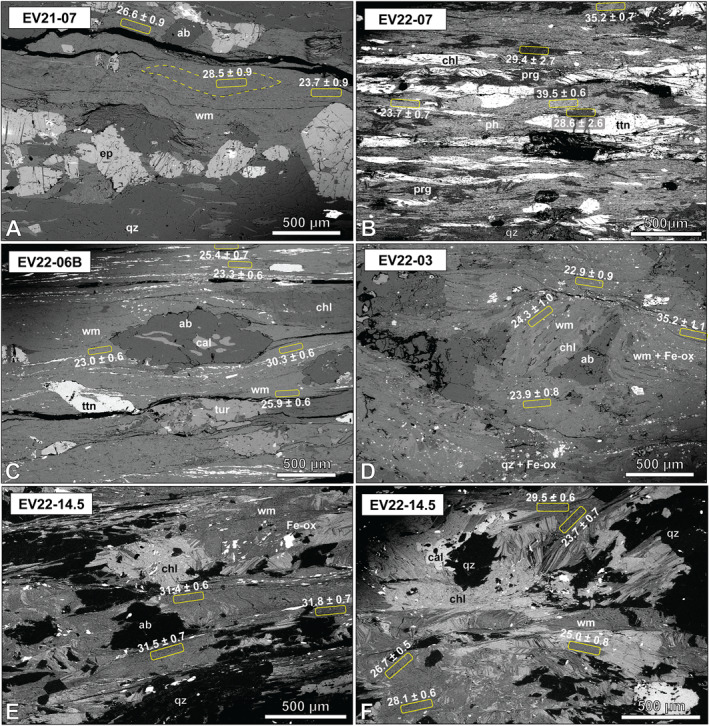
Backscatter electron images showing locations of in situ ^40^Ar/^39^Ar spot analyses. (a) High‐angle white mica “fans” preserves late Oligocene dates, whereas foliation‐defining mica records late Oligocene to early Miocene dates. (b) Discrete patches of phengite and paragonite in EV22‐07 defining a single foliation yield late Oligocene dates within error of one another. Phengite in high‐angle fans yields late Eocene dates. (c) Strain shadow preserving and early Oligocene date among foliation‐defining white mica recording late Oligocene to early Miocene dates. (d) Coarse, blocky mica in EV22‐03 overgrowing albite yield late Oligocene to early Miocene dates. Foliation in this sample has disseminated Fe‐oxides and produces scattered late Eocene and older dates. (e, f) Sample EV22‐14.5 exhibits mica microlithons with a spaced crenulation cleavage. Crenulated mica yields early to late Oligocene dates, whereas mica neoblasts intergrown with chlorite provided late Oligocene to early Miocene dates.

The aggregate in situ ^40^Ar/^39^Ar data display a prominent peak at c. 25 Ma, along with an older shoulder defined by dates between c. 30 and 35 Ma (Figure 10). A limited number of older, middle Eocene to Paleocene dates are also measured in four samples, but represent the dominant age population in only one sample (EV20‐14). Some samples show a correlation between microstructure and ^40^Ar/^39^Ar date. Common relationships of this type include: (a) coarse intrafolial mica “fans” record alternately older or younger ages relative to a planar foliation (Figures 11a and 11b); (b) older dates infrequently recorded by mica occupying strain shadows of albite, epidote, or titanite porphyroblasts (Figure 11c); (c) coarse mica crystals in veins or replacing albite provide younger dates than mica within the foliation (Figure 11d); and (d) crenulated older foliation, including both the relic folded foliations and the spaced cleavage domains, record older dates relative to comparatively undeformed mica crystals defining high angles to the crenulation cleavage or intergrown with chlorite parallel to the cleavage (Figures 11e and 11f).

### 6.2 In Situ ^87^Rb/^87^Sr Geochronology

**Figure 12:**
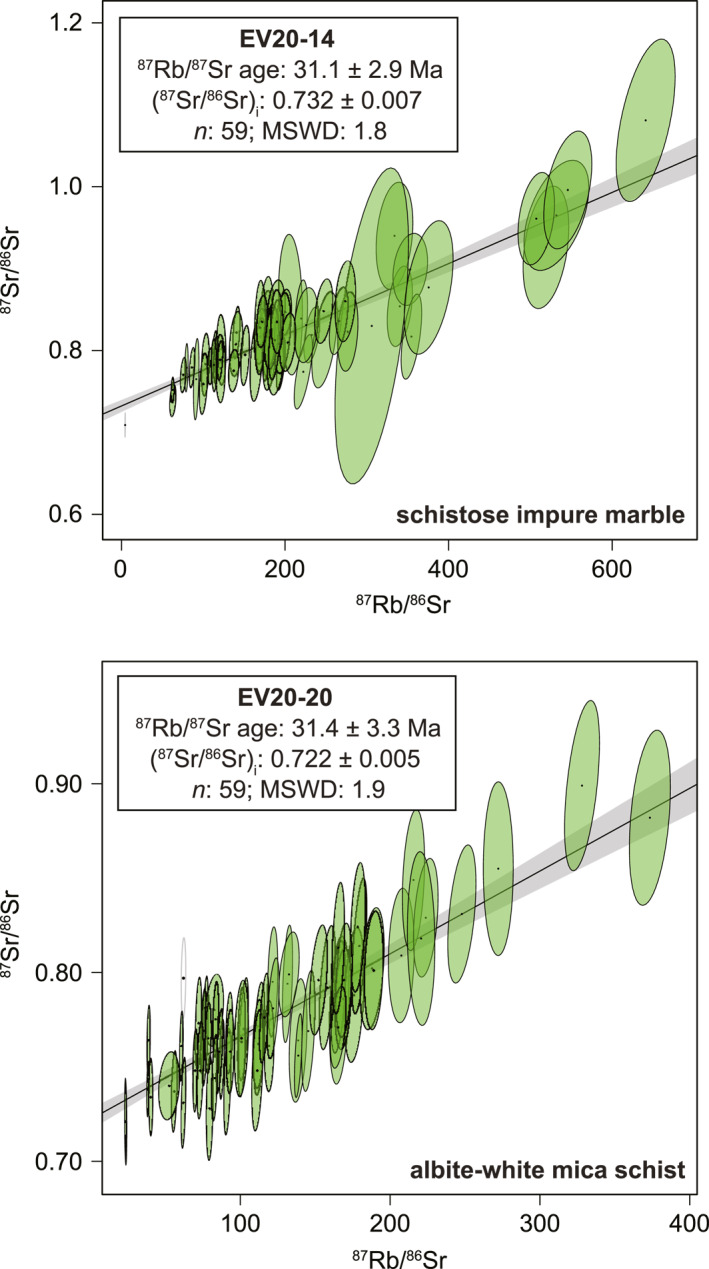
Isochron diagrams calculated using in situ white mica ^87^Rb/^87^Sr geochronology from samples EV20‐14 and EV20‐20. The two isochron dates are indistinguishable, despite the samples yielding contrasting ages via the in situ ^40^Ar/^39^Ar method (see Figure 10). Ellipses are 2*σ* error. Empty ellipses are analyses that were excluded from the isochron regression.

Two samples (EV20‐14, EV20‐20) from among those yielding Eocene and older in situ ^40^Ar/^39^Ar dates were selected for in situ ^87^Rb/^87^Sr geochronology. Sixty spot analyses were obtained from white mica in each sample. Supplemental analyses of low‐Rb phases (calcite, albite) yielded poor analytical results and were excluded from isochron calculations. The complete analytical results are presented in Table S3. The data produce single population regressions that define overlapping isochrons of 31.1 ± 2.9 Ma (EV20‐14; n: 59) and 31.4 ± 3.3 Ma (EV20‐20; n: 59), respectively (Figure 12). Furthermore, the regressions define overlapping ^87^Sr/^86^Sr_i_ of 0.732 ± 0.007 and 0.722 ± 0.005.

## 7 Discussion

The field data reported here outline structural complexities of the main tectonic contact of southern Evia. Most previous interpretations considered the CBU and Basal Unit, constituting the hanging wall and footwall of the Basal Thrust respectively, to have sustained an effectively identical progressive strain history following thrusting (i.e., subduction of the Basal Unit). More recently, Ducharme et al. (2022) described dominantly planar foliated schists in the Tsakei Unit that they interpreted as relating to the so‐called Evia Shear Zone (ESZ), a major structure assisting in unroofing the Basal Unit. Structures within the ESZ were consistent with top‐to‐NE general flattening, implying an oblate finite strain ellipsoid. Although our structural observations are broadly consistent with that finite strain geometry, the Tsakei and flysch units are not characterized solely by planar structures, but instead preserve two orthogonal fold generations (F_1_ and F_2_). A generally W‐dipping axial plane cleavage (S_2_) related to F_2_ is apparent at uppermost structural levels or in quartz‐ or carbonate‐rich lithotypes interspersed with the pelitic schists. High angles between both fold axes and axial planes of the two fold generations would, in principle, yield type‐II refold structures. Along with the dominant easterly vergence of F_2_, the described structures strongly resemble the nappe‐scale folding documented in the Basal Unit marbles at deeper structural levels of the Almyropotamos tectonic window (Ducharme et al., 2022). We consider these structures equivalent to the intrafolial (F_1_) and inclined W‐dipping (F_2_) folds documented near the tectonic contact with the overlying Styra marbles due to their similar orientations, relationships to foliation, and the relative ages.

Hence, the shared (re‐)fold geometry of the marbles in the footwall and the Tsakei Unit near the tectonic contact with the CBU provides, in our view, additional compelling evidence that the Tsakei schists belong to the Basal Unit. This is in accordance with observed similarities in lithologic composition between the Tsakei schists and the Basal Unit flysch succession described both here and previously (Ducharme et al., 2022; Shaked et al., 2000). Elsewhere in the flysch and Tsakei units, a variably spaced to penetrative, shallowly dipping cleavage deforms and crenulates the earlier tectonic fabric that was not documented in the structurally deepest marbles (Figures 4, 5; Ducharme et al., 2022). The resulting folds are close to isoclinal and, unlike the similarly shallow‐dipping F_0_, are not intrafolial (Figures 3, 6 and 6c). Where this cleavage defines a penetrative (or nearly penetrative) foliation, quartz veins exhibit: (a) foliation‐parallel and, less frequently, foliation‐oblique boudinage when oriented at shallow angles to the fabric; and (b) close to isoclinal and locally ptygmatic folding about axial planes parallel to that foliation when cross‐cutting the foliation at high angles (Figure 7). Both structures are locally preserved in alternating hinges and limbs of folded veins, with folded hinges and pinch‐and‐swell or boudinaged limbs developed during their progressive transposition toward the fabric attractor. As those latest fold axes (F_3_) are parallel to the regional and local NE‐SW stretching lineation (Figure 4b), elongation of vein segments in the folded limbs indicates *Y‐*direction stretching that likely also produced the flat‐lying axial plane cleavage and the folds themselves, as previously concluded for similar structures by Ducharme et al. (2022).

Variable intensity of finite strain recorded by various flysch lithotypes, exemplified by variable spacing of F_3_ axial plane cleavage, implies the phyllosilicate‐rich schists accommodated the bulk of the flattening strain. Pre‐existing foliations and folds were fully transposed into a subhorizontal penetrative foliation in these mica and chlorite schists, whereas quartz‐ and carbonate‐rich schists and marbles retain a partial or complete record of the older (i.e., F_1_ or F_2_) structures. Partitioning of finite strain among different lithotypes of the rheologically heterogeneous flysch resembles that described for block‐in‐matrix‐type subduction zone mélanges (Beall et al., 2019; Fagereng & Sibson, 2010; Raimbourg et al., 2019), with finite strain related to the ESZ accommodated dominantly by pelitic schists. Structural depth appears to impose an additional control, as attested to by the local absence of S_3_ near the structural top of the flysch unit. Thus, strain associated with flattening and development of S_3_ in the flysch may intensify down‐section, an apparent departure from the progressive up‐section narrowing and embrittling strain localization expected of a classical detachment (e.g., Lister & Davis, 1989). We hereafter assess the compatibility of our new quartz petrofabric and geochronology data sets with this finite strain model.

### 7.1 Vein Microstructure and c‐Axis Distributions

Deformed veins on southern Evia exhibit quartz microstructures typical of deformation and recovery at moderate temperatures, with only modest local overprinting by lower temperature microstructures (Figure 7). Half of the vein samples (VS‐06D, VS‐06.5A, VS‐07B, VS‐10J, VS‐11F, VS‐16C) contain ribbon grains, some with aspect ratios as high as 10:1. These large (>0.5 mm) left‐over quartz ribbons are mantled by finer grains (with long axes up to 200 μm) of equal size to subgrains developed within the ribbon porphryoclasts, indicative of dynamic recrystallization by subgrain rotation (SGR; Stipp et al., 2002) or Regime II of Hirth and Tullis (1992). Locally, these grains may exhibit planar grain boundary triple junctions. The coarse recrystallized grain size (up to 150 μm) implies that recrystallization proceeded at low differential stress (Stipp & Tullis, 2003), whereas the recrystallization mechanism and triple junctions are consistent with temperatures of ∼400–500°C. The latter microstructures imply a period wherein quartz veins remained at those temperatures yet the primary vein material did not accommodate significant progressive strain. Sutured and bulging grain boundaries and deformation lamellae observed in recrystallized grains of some samples likely correspond to later, lower temperature deformation, at ∼300°C (Passchier & Trouw, 2005; Stipp et al., 2002; Trepmann & Stöckhert, 2003; Figure S2 in Supporting Information S1). Such a two‐stage deformation history is consistent with the established tectonothermal framework of southern Evia. Most notably, the temperatures implied by vein quartz microstructures are in good agreement with temperature estimates coinciding respectively with peak (∼400°C; Shaked et al., 2000) and retrograde (∼315°C; Ducharme et al., 2022) metamorphism of the Basal Unit.

Quartz c‐axis distributions from the veins capture a wide variance of textures (Figure 8), from which we discriminate four subtypes of c‐axis distribution. Five of the 12 pole figures (VS‐07B, EV21‐07, EV22‐03, EV22‐07, EV22‐17) exhibit c‐axis maxima concentrated about the Z axis. Four samples (EV20‐03B, VS‐08H, VS‐10J, VS‐16C) define pole figures with prominent Y‐maxima. Two other vein samples (VS‐10J, VS‐16C), both hosted in chlorite schist, exhibit a small circle distribution parallel or oblique to X in addition to the Y‐maximum. Sample VS‐11F defines a single girdle inclined with respect to the YZ plane. The remaining samples (VS‐06D, VS‐06.5A) show point maximum oblique to the X and Z axes. Quartz c‐axis distributions concentrated about the Z axis, like those in the first group, are generally associated with low‐temperature (∼300°C) deformation exploiting the basal <a> slip system of quartz, although the role of this slip system is contested (Kilian & Heilbronner, 2017). By contrast, c‐axis maxima distributed about Y are inferred to capture prism <a> slip active at more moderate temperatures (∼400°C; Schmid & Casey, 1986). Pole figures generated from vein c‐axis distributions indicate that both regimes are recorded by the veins hosted within the flysch of southern Evia.

Among the samples possessing c‐axis data distributed about the Z axis, three (EV22‐03, EV22‐07, EV22‐17) define small circle girdles centered on Z. The remaining two samples, VS‐07B and EV21‐07, exhibit an asymmetric, type‐I cross‐girdle distribution and a small circle girdle distribution joined by an off‐center girdle segment, respectively. The former textures are generally associated with coaxial flattening (Schmid & Casey, 1986; Sullivan & Beane, 2010), although sample EV22‐07 exhibits a point density asymmetry suggesting a component of non‐coaxial deformation. The latter two may indicate non‐coaxial plane strain deformation (VS‐07B) and deformation between plane strain and flattening (EV21‐07).

Two of the four samples showing Y maxima (EV20‐03B, VS‐08H) define a single point distribution with minor peripheral scatter. The other two Y‐centered samples also define “wings” extending either parallel to (VS‐10J) or oblique to (VS‐16C) the XY plane. The latter samples are hosted in chlorite schist, and similar c‐axis distributions have been interpreted elsewhere as recording progressive rotation of quartz toward the direction of maximum finite stretch without recrystallization (Larson et al., 2014) within the weak chloritic matrix. Moreover, both samples exhibit poor interconnectivity of the quartz aggregates within the partially calcitic vein fill, and thus rigid rotation of quartz aggregates within the less competent carbonate vein fill appears to be a plausible explanation.

Shear sense coinciding with deformation may be inferred either from monoclinic symmetry of the c‐axis distributions, or from monoclinic symmetry with respect to c‐axis density. Among 12 veins analyzed, only four define pole figures that exhibit clear monoclinic symmetry. Samples VS‐11F and VS‐06.5A both record apparent top‐to‐W shear sense, with VS‐11F defining a single inclined girdle, whereas VS‐06.5A defines a clear asymmetric type‐I cross girdle and similarly shows asymmetric c‐axis density distribution. Samples VS‐07B (silicate‐carbonate schist) and EV22‐07 (paragonite‐tourmaline schist) are different lithotypes spaced only several meters apart, and each records a point density asymmetry consistent with top‐to‐E shear sense. Sample VS‐07B also defines a type‐I asymmetric cross‐girdle, although the orientation of the central girdle segment is ambiguous. We also stress that mesoscopic shear sense documented within the ESZ is consistently top‐NE. Thus, whereas the apparent opposing shear sense indicated by some c‐axis textures may be preserved from earlier deformation, they may alternatively be explained as recording local antithetic shearing during coaxial stages of the progressive deformation, or as having been rotated from an initially synthetic sense during folding of vein segments.

We interpret the quartz c‐axis results detailed above as capturing two distinct phases of deformation. Vein samples yielding Y‐centered, c‐axis distributions likely record deformation at moderate temperatures of roughly 400–500°C, whereas samples that provide Z‐centered textures likely record deformation at lower temperatures closer to 300°C. These approximate temperatures are in good agreement with those estimated to coincide with peak HP‐LT metamorphism of the Basal Unit (Shaked et al., 2000) and its subsequent greenschist facies overprint during exhumation (Ducharme et al., 2022; Katzir et al., 2000). The temperatures are also generally consistent with quartz microstructures in the samples. Veins that record pronounced Y‐maxima preserve coarse monocrystalline ribbon grains mantled by quartz showing evidence of dynamic recrystallization by subgrain rotation, implying that these capture the hotter interval of deformation. Most other samples which yield prominent Z maxima lack monocrystalline ribbon grains, signifying either that those veins post‐date the moderate temperature event or that evidence of that event has been fully obscured by later deformation at lower temperatures. The exception to this is sample VS‐07B, which retains ribbon grains and dynamic recrystallization microstructures consistent with the hotter deformation event despite its Z‐maximum and apparent top‐to‐E shear sense. It is noteworthy that both samples whose quartz c‐axis textures indicate apparent top‐to‐W shear sense (VS‐06.5A, VS‐11F) exhibit microstructures consistent with the hotter deformation event, whereas those recording top‐to‐E shear sense (VS‐07B, EV22‐07) instead display maxima about Z and, in the case of EV22‐07, lack quartz microstructures consistent with the hotter deformation. Moreover, whereas flattening strain is evident in structural relationships between veins and the host foliation (e.g., Figure 6c; see also Ducharme et al., 2022), the veins likely underwent rotation during foliation development. Thus, the relatively limited record of deformation related to the syn‐flattening S_3_ foliation is not unexpected, and the pole figures presumably principally capture deformation related either to burial or early exhumation.

### 7.2 In Situ White Mica Geochronology

Considerable geochronological data are now available for the Basal Unit on southern Evia (Ducharme et al., 2022; Maluski et al., 1981; Ring & Layer, 2003; Ring & Reischmann, 2002; Ring, Glodny, et al., 2007; Ring et al., 2001). Most white mica ^40^Ar/^39^Ar and ^87^Rb/^87^Sr dates from the Basal Unit are between c. 35 Ma and c. 21 Ma. Data from exposures of the Basal Unit on Samos and Tinos yield comparatively restricted dates between c. 27 and 21 Ma (Avigad & Garfunkel, 1989; Bröcker & Franz, 1998; Ring & Layer, 2003; Ring & Reischmann, 2002; Ring, Laws, & Bernet, 1999), although whether Tinos exposes the Basal Unit has since been disputed (Bröcker & Franz, 2005). These results have been interpreted either to capture early Miocene under‐thrusting and HP‐LT metamorphism of the Basal Unit (Gerogiannis et al., 2021; Ring & Layer, 2003; Ring, Glodny, et al., 2007), or as recording an Oligocene HP event followed by Miocene greenschist facies metamorphism and extension (Ducharme et al., 2022; Shaked et al., 2000).

Our in situ analyses represent the first microstructurally resolved geochronology from the Basal Unit. In situ geochronology can clarify microstructurally controlled variance in regional ^40^Ar/^39^Ar data sets (e.g., Barnes et al., 2023; Cossette et al., 2015; Di Vincenzo et al., 2022; Laurent et al., 2021). Many studies highlight complexities inherent in the ^40^Ar/^39^Ar system attributed to (sub‐)micron scale heterogeneity with respect to recorded ^40^Ar/^39^Ar date, and may result in discordance between the real or interpreted tectonic significance of certain microstructures and the ^40^Ar/^39^Ar date recorded. For instance, in situ ^40^Ar/^39^Ar data from CBU on Syros and Sifnos preserve the complete Eocene to Miocene interval spanning subduction to exhumation under greenschist facies conditions (Laurent et al., 2021). However, Laurent et al. (2021) noted considerable within‐sample variability that correlates more readily with sample petrology and degree of finite strain, implying some sample‐scale equilibration of ^40^Ar/^39^Ar systematics. Applying that observation to the Basal Unit, which altogether lacks pristine HP‐LT assemblages, our new in situ data may be expected to capture either predominantly the timing of retrograde metamorphism, or exhibit complexity corresponding to finite strain recorded by a given sample. Despite lacking expansive tectonic models commensurate with those available for the CBU, the regional framework afforded by those data and the still somewhat limited constraints on the tectonothermal evolution of the Basal Unit and equivalent unmetamorphosed rocks permit certain assumptions for our own interpretations:

1. Peak HP‐LT metamorphism of the Basal Unit is younger than that of the CBU (c. 50 Ma; e.g., Altherr et al., 1979; Maluski et al., 1987; Tomaschek et al., 2003; Laurent et al., 2021; Uunk et al., 2022), and loosely constrained by the nominal early Eocene maximum depositional age of the flysch unit imposed by fossils documented in the underlying marbles (Dubois & Bignot, 1979);
2. The Gavrovo‐Tripolitza carbonate platform collided outboard of the Pindos oceanic suture, which occurred between c. 36 Ma and 29 Ma (Sotiropoulos et al., 2003; Thomson et al., 1998); thus HP metamorphism of the Basal Unit likely coincided with, or shortly post‐dated, this period (while also satisfying [1]);
3. At least on Evia, zircon (U‐Th)/He cooling ages require that the Basal Unit exhumed into the brittle crust (<200°C) before the middle Miocene (c. 17 and 15 Ma; Ducharme et al., 2022), synchronous with the CBU.

Overall, variability in our in situ ^40^Ar/^39^Ar data correlates with host lithotype, cm‐ to m‐scale structures, and position within the structural pile. As expected, microstructural context of the ablated mica correlates inconsistently with the empirical date (Table 1; Figure 11). This may be partially attributable to fine mica grains defining foliation in our samples, which can skew ^40^Ar/^39^Ar ages toward homogeneous younger dates (Barnes et al., 2023; Di Vincenzo et al., 2022). All samples, except for one, consist of phengitic white mica with average Si apfu >3.3, with the two carbonate‐rich samples predictably containing the most aluminoceladonite‐rich mica (>3.5 Si apfu; Cossette et al., 2015; Rogowitz et al., 2015; Table 1, Figure 9). Although the high‐Si white mica records the oldest coherent date populations among our data, most samples show poor correlation between apparent ^40^Ar/^39^Ar age and white mica major element chemistry (e.g., Bröcker et al., 2004; Di Vincenzo et al., 2004; Laurent et al., 2021). Further, ^40^Ar/^39^Ar dates from the two carbonate‐rich samples differ by ∼15 Myr; accordingly, sample structure, not mineral chemistry, offers a more compelling first‐order control.

Perhaps unsurprisingly, broad similarities in both the age populations of our in situ data, and in their spatial distribution within the structural pile of Evia, direct us to similar conclusions as the single‐grain fusion data (Ducharme et al., 2022). Unimodal, late Oligocene to early Miocene age populations dominate mica schist samples from middle and lowermost structural levels of the flysch (EV22‐06B, EV19‐03, EV22‐07), and in one mica schist from directly below the tectonic contact with the CBU (EV21‐07). Isolated older dates in these samples are obtained only from microstructures for which they are easily justified, like porphyroblast strain shadows (Table 1; Figure 11). The same microstructure elsewhere may yield dates indistinguishable from foliation‐defining mica, affirming the inconsistent relationship between microstructure and ^40^Ar/^39^Ar systematics (e.g., Laurent et al., 2021).

Sample EV22‐07 is unique in that it contains both phengite and paragonite (Figure 9). The two micas generally occur in distinct clusters. With one exception, phengite defining foliation yields young dates of 21.1 ± 0.8 to 23.7 ± 0.7 Ma, whereas paragonite records slightly older ages of 26.4 ± 1.8 to 31.8 ± 4.4 Ma (Figure 11). Large errors due to the low K of paragonite mean that even the older apparent ages are within 2*σ* error of the complete range of phengite dates (Figure 10), and thus their relative age is indeterminable from our data. However, three high‐angle fans composed of phengite or mixed phengite‐paragonite produced older dates between 35.2 ± 0.7 and 42.0 ± 0.7 Ma, distinctly older than dates obtained from foliation‐defining phengite.

Carbonate‐rich samples from similar structural depths as the samples discussed thus far yielded inconsistent results. One sample (EV20‐14), a schistose impure marble previously dated as late Oligocene by the multiple single‐grain fusion method, yielded by the in situ method a unimodal middle Eocene age population (Ducharme et al., 2022). That discrepancy is discussed further below. The other carbonate‐silicate schist sample (EV22‐14.5) was obtained only ∼10 m above the contact with the Basal Unit marble sequence. Micaceous microlithons in this sample are composed of crenulated phengite defining a spaced, subhorizontal cleavage dissecting an older foliation. Cleavage domains and relict foliation alike yield ages that define a continuous trend, preserving dates approximating the youngest dates in the mica schists (c. 26.7 ± 0.5 Ma), increasing toward a maximum captured by five analyses at c. 32 Ma (Figure 10).

The remaining three samples, all from near the structural top of the flysch unit, provided comparatively scattered ^40^Ar/^39^Ar dates. Uniquely among these, sample EV20‐20 records a relatively unimodal late Eocene age population within error of its single‐grain fusion dates (Ducharme et al., 2022). The other two samples retain the youngest late Oligocene dates (EV22‐03, EV22‐17), recorded by blocky white mica overgrowing albite porphyroblasts or in veins (Figure 11). The mica appears to post‐date initial vein sealing, as the host veins have been boudinaged and rotated into the foliation. Foliation‐defining mica in these samples conversely records generally dispersed dates as old as early Eocene, with limited Paleocene dates in one sample. Those analyses, as well as all data from sample EV20‐14, provide dates older than the earliest proposed timing of metamorphism for the Basal Unit (c. 35–30 Ma). These older dates have three possible origins: (a) unsupported ^40^Ar; (b) detrital grains whose ^40^Ar/^39^Ar systematics were not reset during metamorphism; or (c) a hitherto undocumented earlier (pre‐late Eocene) tectonic event affecting the Basal Unit.

To further clarify the ^40^Ar/^39^Ar results, we performed in situ ^87^Rb/^87^Sr dating of white mica in two samples yielding moderately dispersed, late Eocene and older dates. For EV20‐20, which yielded an ^87^Rb/^87^Sr isochron date of 31.4 ± 3.3 Ma, three dating methods using two isotope systems (single‐grain fusion ^40^Ar/^39^Ar, in situ ^40^Ar/^39^Ar, in situ ^87^Rb/^87^Sr) now affirm that white mica in the sample records an event of latest Eocene to earliest Oligocene age. Notably, the weighted mean of total fusion dates is older and outside of error of the two in situ analyses; however, this may be a consequence of inclusions, which were deliberately avoided during in situ analysis but may have been unavoidable during grain separation for total fusion.

Conversely, EV20‐14 has now provided three apparently distinct ages. With respect to the ^40^Ar/^39^Ar data, white mica analyzed by single‐grain fusion produced better radiogenic ^40^Ar yields than those analyzed in situ (Table 1; cf. Table S3 of Ducharme et al., 2022), perhaps representing two distinct mica populations. Despite deliberately targeting the same mica populations, the in situ ^87^Rb/^87^Sr and ^40^Ar/^39^Ar data sets still produced different dates.

Although the ^87^Rb/^87^Sr system often behaves similarly to the ^40^Ar/^39^Ar system (Bosse & Villa, 2019; Halama et al., 2018), it is rarely subject to addition of daughter isotope and consequent obscuring of geologically meaningful dates (Gyomlai et al., 2023; Larson et al., 2023). This distinction is well illustrated by the two samples analyzed herein, which produced indistinguishable ^87^Rb/^87^Sr isochron ages of 31.1 ± 2.9 Ma and 31.4 ± 3.3 Ma (Figure 12). The presence of unsupported ^40^Ar is typically inferred by comparison with other radiometric dates and by assessing geological plausibility (e.g., Warren et al., 2012). Whereas the ^87^Rb/^87^Sr data from EV20‐14 are younger than ^40^Ar/^39^Ar dates from the sample, the relationship usually observed when unsupported ^40^Ar is present (Larson et al., 2023), the relatively unconstrained maximum depositional age (“post‐early Eocene”) of the Basal Unit flysch do not unequivocally support this interpretation of the middle Eocene and older ^40^Ar/^39^Ar dates. However, we note that scattered Eocene in situ ^40^Ar/^39^Ar dates were measured in mica deformed within prominent Cʹ‐type shear bands in sample EV22‐17 (Table 1). These structures should occur relatively late in the ductile strain history of the Basal Unit, likely in the Oligocene (Ducharme et al., 2022). Despite evident strain imposed during formation of the shear bands, deformed mica within the shear bands records dispersed dates, including some older than those obtained from the main foliation. In our view, this dispersion is best explained by unsupported ^40^Ar, at least for EV22‐17.

In summary, our new in situ geochronology can be generalized as capturing two geologically significant dates. The first, of apparent early Oligocene age, is evident in ^40^Ar/^39^Ar data from samples EV20‐20 and EV22‐14.5, and captured by both ^87^Rb/^87^Sr isochron dates. Isolated microstructural features, like albite strain shadows in EV22‐06B, may also preserve this signal. A second, younger late Oligocene to early Miocene age signature is prevalent in samples exhibiting a coherent, planar foliation. The key distinction between samples preserving one population of ^40^Ar/^39^Ar dates appears to be of a structural nature. Samples preserving the older dates exhibit incomplete transposition of older folds and foliations, whereas those with an outwardly simple structure likely represent, as surmised in preceding discussions, tectonic fabrics newly transposed following flattening and exhumation. Sparse, older ^40^Ar/^39^Ar dates cannot be discounted as geologically meaningless, and may capture preservation of pre‐metamorphic detrital grains or a yet undocumented earlier metamorphic event affecting the Basal Unit. Nevertheless, due to the good adherence to the proposed timing of subduction of the Basal Unit protoliths (Sotiropoulos et al., 2003; Thomson et al., 1998), previous documentation of detrital mica grains in the flysch (Ducharme et al., 2022), as well as microstructural context and ^87^Rb/^87^Sr data which do not preclude the presence of unsupported ^40^Ar, we remain skeptical of the geological significance of the older Eocene dates. We instead interpret the early Oligocene dates as reflecting the timing of burial and HP‐LT metamorphism of the Basal Unit, as the dates are evident mainly where the foliation deformed by F_2_ folds (e.g., EV20‐20) or in crenulation domains between non‐penetrative examples of the S_3_ cleavage (e.g., EV22‐14.5). We interpret the younger late Oligocene dates as dating deformation related to the ESZ and consequent exhumation, as the dates dominate in samples showing a single coherent S_3_ foliation. These conclusions are broadly aligned with previous models positing maximum burial of the Basal Unit in the Oligocene (Ducharme et al., 2022; Maluski et al., 1981; Shaked et al., 2000).

### 7.3 Superimposed Records of Contraction and Tension

The structural record of HP‐LT units in the Cyclades represents a protracted interval of contraction followed by regional scale tension. Contractional structures encompass those produced during subduction and subsequent syn‐orogenic wedge extrusion, whereas tensional structures are dominantly those related to ductile‐then‐brittle low‐angle detachment faulting and younger, brittle high‐angle normal faults. All but the high‐angle faults operated initially under ductile conditions and have thus produced a complex array of structural relationships ranging from total obfuscation of earlier structures to partial overprinting or geometric modification (Ring et al., 2010). In the Cyclades, the spatial distribution of major post‐orogenic structures subordinate to the NCDS and WCDS remains incompletely understood (Bakowsky et al., 2023; Coleman et al., 2020; Huet et al., 2009; Schneider et al., 2018). Without a clear and diagnostic deformation structures, like the knife‐sharp fault planes typical of detachments, there are few methods by which to unequivocally distinguish a structure that operated during contraction from another that operated under tension. Several important structures in the Cyclades exemplify such, oftentimes unresolved, controversies (e.g., Ios, Forster & Lister, 1999; Huet et al., 2009; Syros, Trotet et al., 2001; Philippon et al., 2011; Laurent et al., 2016).

The tectonic contact exposed on southern Evia encapsulates one further example of this debate. We contend that available geochronology all but requires that the Basal Unit flysch delineates an important tectonic boundary across which significant exhumation has occurred, and that this conclusion is supported by the new structural observations outlined here. Effectively indistinguishable ZHe dates measured throughout the structural pile demonstrate significant attenuation of intervening levels between the CBU and Basal Unit prior to their eventual exhumation into the brittle crust (Ducharme et al., 2022). The white mica ^40^Ar/^39^Ar data point to much the same conclusion. Foliation‐defining white mica with a demonstrable relationship to stretched and transposed quartz veins consistently yields the youngest ^40^Ar/^39^Ar dates documented on Evia (Ducharme et al., 2022; Maluski et al., 1981; Ring & Layer, 2003; Ring, Glodny, et al., 2007; this study). The coexisting structures suggest that the young dates capture ductile strain‐induced resetting of the ^40^Ar/^39^Ar systematics during ductile thinning of the flysch, providing a mechanism to directly explain the ZHe data.

Here, we have newly shown that this late Oligocene age signature is resolvable at all structural levels of the Basal Unit flysch. Furthermore, our in situ ^40^Ar/^39^Ar and ^87^Rb/^87^Sr data provide the first microstructurally resolved geochronology for the Basal Unit. The structural data and geochronology together yield an internally consistent, time‐resolved progressive strain history for the ESZ. Puzzlingly, despite considerable geochronological evidence that the ESZ accomplished significant exhumation of the Basal Unit, its strain geometry differs considerably from any equivalent structure documented in the Cyclades. The mechanical influence of these major structures is indicated in most instances by a wide zone of intense mylonitization imposed upon their footwalls. On the other hand, a considerable volume of material at any given structural position within the ESZ shows little evidence of having accommodated the intense flattening observed in the pelitic lithotypes. The ^40^Ar/^39^Ar systematics of mica in the footwalls of most major detachments in the Cyclades are comprehensively reset, and now date the timing of mylonitization (Grasemann et al., 2012; Jolivet et al., 2010). Meanwhile, the ESZ, throughout its thickness, preserves blocks in which earlier portions of the progressive strain history and corresponding age signature remain essentially intact.

The Mesochoria cataclasites (Figure 2) imply that the tectonic contact accommodated some brittle deformation. Finite strain documented in the vicinity of the contact is, nonetheless, atypical in many ways compared to finite strain observed at major tectonic boundaries elsewhere in the Cyclades. First, although the Mesochoria cataclasites extend along several hundred meters of strike length, further lateral continuity was either not observed or else too poorly exposed, where predicted, for conclusive correlation. Accordingly, cataclasis of the contact may only be relatively localized. Second, mylonites are neither observed within, or immediately structurally below, the cataclasites, suggesting the tectonic contact lacks part of the characteristic strain geometry classically associated with detachment faulting (Lister & Davis, 1989). Lastly, the non‐mylonitic schists in the immediate footwall of the tectonic contact mark an abrupt ∼90° change in strike. Both the cataclasites and the Styra marbles in the hanging wall dip to the northeast; whereas, rocks structurally below the cataclasites dip to the southwest. The latter two points attest further to the apparently limited mechanical influence of the cataclastic deformation.

Nevertheless, the width of the cataclastic zone (5–10 m) suggests accommodation of non‐negligible, albeit localized, brittle deformation. Moreover, the orientation of the cataclastic horizon, and shear sense indicators preserved within and structurally below it, show that cataclastic deformation acted with kinematics synthetic to those of both the ESZ and the NCDS, and parallel to overall extension in the Aegean. Available geo‐ and thermochronology, compounded by the new data presented here, requires that the cold, brittle cataclasites are no older than early or middle Miocene in age, redoubling the likelihood of a relationship to Aegean extension. Integrating this with the flattening and top‐to‐NE simple shear components observed in ductile and brittle‐ductile strain related to the ESZ, which Ducharme et al. (2022) noted show a progression from ductile to brittle‐ductile deformation, it is tempting to conclude that the ESZ preserves some expression, however disparate, of the complete ductile‐then‐brittle progressive strain history expected of a major detachment fault.

### 7.4 The Evia Shear Zone: A Unique Cycladic Exhumation Structure?

Evia is situated in the footwall of the NCDS, one of two crustal‐scale detachment systems facilitating post‐orogenic exhumation in the Cyclades (Jolivet et al., 2010). Whereas the structure is projected offshore of Evia, the mechanical influence of the NCDS is evident in top‐to‐NE brittle‐ductile shear bands developed in rocks correlated with the CBU on the island (Ducharme et al., 2022; Jolivet et al., 2004). The NCDS likely accomplished significant unroofing of southern Evia (Jolivet et al., 2010). Although southern Evia marks the northwestern limits of continuous exposure of the ACCB, it has been suggested that the NCDS extends along strike as far as the Pelion peninsula (Hinshaw et al., 2024). Conversely, the WCDS exhibits structural evidence consistent with that structure terminating in western Attica, at a similar along‐strike position to where Evia lies along the NCDS (Coleman et al., 2020). At its proposed terminus on Mt. Hymmitos, the WCDS occurs as a pair of co‐active brittle‐then‐ductile detachments. Whereas the upper detachment divides Upper Unit (Pelagonian) above from CBU below, as is normally the situation in the Cyclades, Coleman et al. (2020) interpreted the second detachment as juxtaposing two subdivisions of the CBU, structurally below the first.

**Figure 13:**
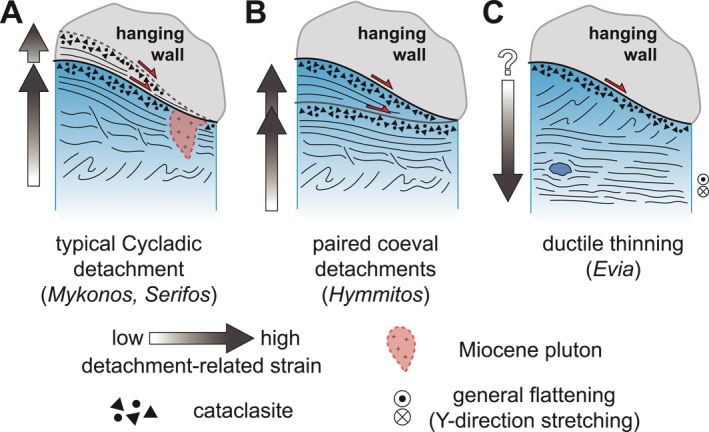
Three types of detachment architecture described from the Cyclades. (a) Typical Cycladic‐style low‐angle ductile‐then‐brittle detachment. Finite strain intensity increases toward the hanging wall, with late cataclasis and knife‐sharp faulting localized at the upper contact. A second, younger ductile‐then‐brittle detachment develops due to heating from syn‐tectonic intrusions. Detachment architecture described in Jolivet et al. (2010) and Grasemann et al. (2012). (b) Paired co‐active detachment branches like those described at Mt. Hymmitos. Detachment architecture develops coevally, with each exhibiting its own ductile‐then‐brittle finite strain evolution. Described in Coleman et al. (2020). (c) Detachment architecture as described in this study for the Evia Shear Zone, with a down‐section intensification of ductile finite strain, involving a flattening component, and cataclastic deformation localized along the upper contact with the hanging wall. Competent phacoids record lower finite strain than the surrounding matrix.

Several compelling parallels may be drawn between the ESZ and the Hymmitos detachments. First, the ESZ developed structurally below the extensional contact between the Upper Unit and CBU, in contrast to more classical examples from the Cyclades that branched structurally upward (Jolivet et al., 2010; Lecomte et al., 2010; Lister & Davis, 1989; Figure 13a). Second, geochronological data suggest ductile strain was accommodated synchronously along the ESZ and NCDS, as described for the Hymmitos detachments along the WCDS (Figure 13b), also in contrast to other branched Cycladic detachments (Coleman et al., 2020; Ducharme et al., 2022; Jolivet et al., 2010). Thermochronology collected astride the lower detachment of Hymmitos indicates diachronous exhumation of its hanging wall and footwall; across the ESZ, meanwhile, ZHe dates from the Basal Unit and CBU are identical (Coleman et al., 2020; Ducharme et al., 2022). Additionally, unlike the strain distribution described in the preceding section for the ESZ (summarized in Figure 13c), the lower detachment of Hymmitos still exhibits the conventional strain geometry of a low‐angle normal fault. The ESZ may thus be responsible only for juxtaposing the Basal Unit and CBU at similar structural levels, whereas the NCDS accommodated exhumation of both nappes into the upper crust since the early Miocene.

It is relevant to note that many of our underlying assumptions are based on the age of the Basal Unit marbles, which are established from fossils as Lower to Middle Eocene in age (Dubois & Bignot, 1979). Subsequent research has, perhaps erroneously, imposed a Middle to Upper Eocene age for the overlying flysch (Ducharme et al., 2022; Ring, Glodny, et al., 2007; Shaked et al., 2000), despite no direct evidence supporting such an age constraint. If the flysch were Upper Eocene in age, the sparse Middle Eocene ^40^Ar/^39^Ar dates (Figure 10) may represent a geologically plausible early metamorphic event rather than contamination by unsupported ^40^Ar. To a further extreme, the fossil observations on which the interpreted age is based have, to our knowledge, not been reproduced since, and recent popular interpretations depict the CBU as constructed of several shear zone‐bounded nappes (e.g., Kotowski et al., 2022; Peillod et al., 2024; Uunk et al., 2022), drawing a potentially compelling analogue with the two HP‐LT nappes of southern Evia. Although we acknowledge both alternative scenarios as viable, we are likewise reluctant to misrepresent the fossil observations of Dubois and Bignot (1979). We defer to evidence from the External Hellenides, which similarly supports a late Eocene or early Oligocene initiation of subduction for the Basal Unit (Sotiropoulos et al., 2003; Thomson et al., 1998), and maintain the interpretations of our new geochronology as discussed in Section 7.2.

**Figure 14:**
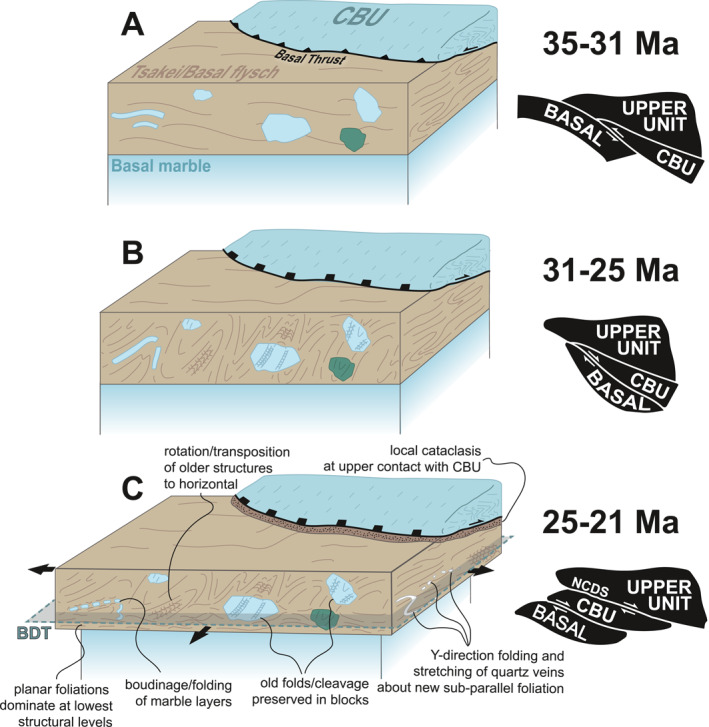
Schematic 3D block diagram and related cross‐section model for the tectonic evolution of the Evia Shear Zone from subduction thrusting of the Basal Unit (a), syn‐orogenic extrusion (b), and post‐orogenic unroofing and coaxial flattening (c). Basal Unit flysch within the Evia Shear Zone records syn‐convergence fabrics and structures that were partially, or locally totally, overprinted during exhumation by structures related to flattening and consequent vertical ductile thinning. See text for details.

Notwithstanding the limited data constraining the geodynamic history of the Basal Unit, its participation within the broader evolution of the Aegean Sea can be inferred using the more established framework of the CBU. Peak HP metamorphism recorded by the Basal Unit only reached ∼10 kbar and ∼400°C, slightly lower than peak metamorphism recorded by the CBU on Evia (Shaked et al., 2000). It can be assumed that these metamorphic maxima were achieved diachronously, as the CBU was likely undergoing syn‐orogenic wedge extrusion as the Basal Unit was subducting in the late Eocene (Huet et al., 2015; Jolivet et al., 2010; Laurent et al., 2016; Peillod et al., 2024; Ring, Glodny, et al., 2007; Xypolias et al., 2003). Consistent with previous models, this interval of syn‐orogenic unroofing of the CBU coincides with subduction of the Basal Unit, juxtaposing the two along the Basal Thrust (e.g., Gerogiannis et al., 2021; Ring, Glodny, et al., 2007; Xypolias et al., 2003; Figure 14a). Based on inferred rapid exhumation rates, Ducharme et al. (2022) similarly proposed an interval of syn‐orogenic unroofing of the Basal Unit. The timing of this stage remains unclear, and syn‐orogenic exhumation may have initiated immediately after peak metamorphism at c. 32 Ma, or else at any time before c. 25 Ma (Figure 14b). By 25 Ma, pre‐existing folding (i.e., F_0_, F_1_) and related structures were being transposed and overprinted by a sub‐horizontal cleavage (S_2_) of variable intensity related to vertical flattening (Figure 14c). At this time, metamorphic assemblages developed in rocks exhibiting penetrative sub‐horizontal foliations and macroscopic structural evidence of flattening strain developed under lower greenschist facies conditions at somewhat elevated pressures (310 ± 15°C, 7 ± 1 kbar; Ducharme et al., 2022). Note that this estimate is obtained from the base of the schists. If the Basal Unit flysch and Tsakei schists are, as suggested here, laterally equivalent—that is, they do not contribute cumulatively to the thickness of the structural pile as depicted in Katsikatsos (1991a)—then the total measured thickness of the schists is ∼1.5 km. Assuming modest attenuation of ∼25% due to ductile thinning (e.g., Ring & Kumerics, 2008; Xypolias et al., 2010), and a moderately relaxed post‐subduction regime geotherm of 20°C/km, this would yield a ∼2 km thick restored section and a vertical temperature differential of ∼40°C between structurally highest and lowest schists. At temperatures as low as those estimated, parts of the structural pile would be approaching the brittle‐ductile transition for white mica (∼250°C; Akker et al., 2021; Dunlap et al., 1991; White, 2001), whereas mica at lower structural levels would still be deforming via fully ductile mechanisms. Conversely, some sections of the Basal Unit flysch are fewer than 500 m thick, implying either significant variability in primary depositional volumes, lateral changes in intensity of ductile thinning, or a combination of the two. Our data do not allow us to attribute the thickness variations to either cause, and detailed mapping of the flysch interval would help to clarify this.

Contrasts in strain partitioning behavior linked to lithology are documented elsewhere, with similar consequences for the ^40^Ar/^39^Ar record of white mica (Beaudoin et al., 2020; Cossette et al., 2015; Laurent et al., 2021). Conversely, the apparent down‐section intensification of finite strain observed in the schists implies a strain gradient not generally predicted for extensional structures, which produce footwall strain that intensifies up‐section toward the detachment plane (Grasemann et al., 2012; Lister & Davis, 1989). It is also inconsistent with observations of shear zone evolution more generally, which record the highest finite strain typically in their centers (Fossen & Cavalcante, 2017). In the Cyclades, mylonites in the footwall of the major detachments often deform coherent marbles and mica schists of the CBU, or the late Miocene granitoids that intrude them (Grasemann et al., 2012; Jolivet et al., 2010). In this way, the heterolithic composition of the Basal Unit flysch may differ significantly, and behave more similarly to a block‐in‐matrix mélange (Beall et al., 2019; Fagereng & Sibson, 2010). Bulk deformation of these rocks typically proceeds with strain partitioning strongly into the weak matrix, with the more competent clasts and blocks accommodating comparatively minor finite strain (Fagereng & Sibson, 2010). This prediction aligns well with our empirical field observations, in which overprinting of older structures is most severe in the weak pelitic matrix, but comparatively minor in the other constituent lithotypes.

Detachment faults commonly localize along major pre‐existing discontinuities, like nappe boundaries, but local geology may cause structures to deviate from this rule. On Tinos and Mykonos, intrusion of middle Miocene granitoid magmas upwarped the local geotherms, causing new detachment branches to form structurally above the main branch, but below the displaced brittle‐ductile transition (Jolivet & Patriat, 1999; Jolivet et al., 2010; Lecomte et al., 2010). On Folegandros, progressive strain localized at the center of the island shows a progression from ductile to brittle‐ductile structures, but a brittle detachment plane is situated at higher structural levels in the hanging wall, with little related strain apparent at intervening levels (Bakowsky et al., 2023). A similar migration in the locus of deformation may explain the structures observed in the Basal Unit flysch on Evia. Strain remained localized near the basal contact with the Basal Unit marbles for as long as the structurally lowest schists were able to accommodate it via ductile mechanisms. More competent lithologies, like carbonate and quartzose schists and quartzites, sustained less intense deformation, and at higher structural levels still preserve minimally reworked older subduction‐related structures and foliations. Once the schistose package had fully exhumed above the brittle‐ductile transition for micaceous rocks, distributed deformation became unfavorable, and strain instead localized as fully brittle cataclasis along the structurally higher tectonic contact with the CBU (Figures 2 and 13c). Mounting evidence, even in the Cyclades alone, indicates that detachment faults are highly sensitive to bulk rheology, as influenced by prevailing PT conditions and lithological context, during their activity. Low‐angle normal faulting within the rock record may therefore be expected to exhibit a wide variability in expression that is intimately tied to its immediate geological context. Despite potentially facilitating considerable magnitudes of exhumation, some of these structures may be obscured by meter‐scale heterogeneities that display an apparently incoherent record of extensional deformation.

## 8 Conclusions

The uppermost structural levels of the Basal Unit on southern Evia comprise a lithologically heterogeneous schistose metasedimentary succession, interpreted as a flysch package, that experienced Cenozoic HP‐LT metamorphism. This interval of rock divides two marble‐dominated units that represent two distinct thrust sheets in the broader Hellenic orogen, the Basal Unit and the Cycladic Blueschist Unit. The current exposure of this syn‐convergent thrust has been re‐exploited or overprinted by diffuse extensional strain accommodated primarily by the intervening package of flysch. The resultant structure, the Evia Shear Zone, drove unroofing of the Basal Unit marbles, exhuming them into the brittle crust simultaneously with the CBU hanging wall.

New in situ white mica ^40^Ar/^39^Ar and ^87^Rb/^87^Sr geochronology highlight similar predominant age signatures as documented previously from the Basal Unit flysch. Dominant microstructure‐resolved trends indicate the younger late Oligocene age population is recorded predominantly by schists displaying a single planar metamorphic foliation, whereas those preserving a spaced secondary cleavage and older foliation record the older late Eocene dates. Host lithotype exerts a major first‐order control on the differential preservation of relict structural features, a trend which is corroborated by the macroscopic structural record observed in more carbonate‐ and quartz‐rich schists, and in quartz microstructures and c‐axis distributions. The distinct age populations are interpreted to reflect late Eocene‐early Oligocene subduction and HP‐LT metamorphism, followed by recrystallization of mica during ductile strain related to exhumation in the late Oligocene.

Exhumation of the Basal Unit was accomplished in part by general shear localized in the flysch, manifesting with a top‐to‐NE non‐coaxial component, alongside superimposed coaxial flattening. The resulting oblate finite strain ellipsoid is captured in outcrop by structures which demonstrate extension operating parallel to the intermediate stretching (Y‐) axis, such as boudinaged quartz veins. Quartz in the deformed veins yields variable c‐axis distributions, but several nonetheless record *Z*‐centered distributions, including small‐circle girdles, consistent with the flattening strain inferred from macroscopic structures.

Rather than a single continuous ductile‐then‐brittle detachment as is common in the Cyclades, strain in the ESZ partitioned diffusely into more mica‐rich lithotypes, preserving older structures in more competent blocks. The situation described on Evia attests to the diversity of deformation styles capable of facilitating exhumation of HP‐LT rocks. Moreover, the differential record of finite strain produced in the highly rheologically heterogeneous flysch succession parallels observations from similar block‐in‐matrix mélanges commonly found in exhumed subduction zones (Beall et al., 2019; Fagereng & Sibson, 2010). The apparently discontinuous finite strain had effectively obscured the presence of an exhumation‐related structure, and thus a potential role for similar rocks should be re‐evaluated in terranes where magnitudes of unroofing appear to exceed the capacity of previously identified structures.

## Supporting information

Supporting Information S1

Table S1

Table S2

Table S3

## Data Availability

Full data tables referenced in the text additional figures, and detailed methodological descriptions are archived in the Mendeley Data repository accessible at the following link: https://doi.org/10.17632/sh55g2j48n.1.
