# Supplementary material for: Strain Partitioning in a Flattening Shear Zone: Re‐Evaluation of a Cycladic Style Detachment
Source: Tectonics. 2024 Nov 21;43(11):e2024TC008412. doi: 10.1029/2024TC008412 (PMC11582021; doi:10.1029/2024TC008412)
Supplement: Supplementary file 1 — Supporting Information S1 [file TECT-43-0-s003.pdf]

AGU PUBLICATIONS

Tectonics

Supporting Information for

Strain partitioning in a flattening shear zone: re-evaluation of a Cycladic-style detachment

Taylor Ducharme[1], David Schneider[1], Bernhard Grasemann[2], Victoria Scoging[1], Christina Bakowsky[1], Kyle P. Larson[3], Alfredo Camacho[4]

1. Department of Earth and Environmental Science, University of Ottawa, Ottawa, Canada
2. Department of Geodynamics and Sedimentology, University of Vienna, Vienna, Austria
3. Department of Earth, Environmental and Geographic Sciences, University of British Columbia Okanagan, Kelowna, Canada
4. Department of Earth Sciences, University of Manitoba, Winnipeg, Canada

Contents of this file

Text S1 to S2
Figure S1 to S2

Additional Supporting Information (Files uploaded separately)

Captions for Tables S1 to S3

Introduction

The supplementary file contains detailed analytical methodologies and fabric analyzer micrograph scans and supplemental micrographs (**Figure S1** and **Figure S2** below). Tabulated data files for the white mica mineral chemistry (**Table S1**), *in situ* $^{40}Ar/^{39}Ar$ geochronology (**Table S2**), and *in situ* $^{87}Rb/^{87}Sr$ geochronology (**Table S3**), are available in the following Mendeley Data Repository:

https://doi.org/10.17632/sh55g2j48n.1

Text S1. Microanalytical methods

<u>S1.1. Scanning Electron Microscopy (SEM)</u>

Scanning electron microscopy was performed at the University of Ottawa using a JEOL 6610LV SEM. Backscatter electron imaging and exploratory electron dispersive spectroscopy (EDS) were carried out with an accelerating voltage of 15 kV and a working distance of 11 mm on standard 30 μm thick polished thin sections.

S1.2. Electron Microprobe (EMPA)

Mineral chemistry of white mica was analyzed via electron microprobe (EMPA). Analyses were performed at University of Ottawa using a JEOL JXA-8230 SuperProbe. Mica was analyzed using a beam diameter of 10 μm with an accelerating voltage of 15 keV and beam current of 20 nA. Dwell times of 10 s were used for X-ray peak positions and 5 s for background positions. Wavelength dispersive spectroscopy (WDS) acquired quantitative chemical data for the major and minor elements Si, Al, Ca, Mg, Fe, K, Na, Ti, Cr, Mn, Cl, and F. The laboratory standards used at University of Ottawa were sanidine (Si, Al, K), diopside (Ca, Mg), hematite (Fe), albite (Na), rutile (Ti), chromite (Cr), and tephroite (Mn); and at Uppsala University were pyrope (Si, Mg), wollastonite (Si, Ca), Al_2O_3 (Al), MgO (Mg), fayalite (Fe), orthoclase (K), albite (Na), pyrophanite (Ti, Mn), Cr_2O_3 (Cr), apatite (P, F), and vanadinite (F, Cl).

White mica compositions were recalculated manually on the basis of 11 O, following the methodology of Vidal & Parra (2000) and Parra et al. (2002), accepting only analyses with weight percent oxide sums between 92-96% and with < 0.5 wt.% TiO_2 + MnO + CaO. Recalculations assumed 11 oxygens and calculated molar fractions for seven end-members (Mg- and Fe-Al-celadonite, muscovite, annite, phlogopite, paragonite, pyrophyllite). All mineral chemical data and associated formula recalculations may be found in **Table S1**.

Text S2. $^{40}Ar/^{39}Ar$ geochronology

Once samples were chosen, characterized, and chips were prepared, $^{40}Ar/^{39}Ar$ analytical work was performed at the University of Manitoba (Winnipeg, Canada) using a multi-collector Thermo Fisher Scientific ARGUS VI mass spectrometer, linked to a stainless steel Thermo Fisher Scientific extraction/purification line and Photon Machines (55 W) Fusions 10.6 CO_2 laser. Argon isotopes (from mass 40 to 37) were measured using Faraday detectors with low noise 1 x 10^{13} Ω resistors and mass 36 was measured using a compact discrete dynode detector. The sensitivity for argon measurements is ~6.312 x 10^{17} moles/fA as determined from measured aliquots of Fish Canyon Sanidine (Dazé et al., 2003; Kuiper et al., 2008).

Unknowns were placed in 2 mm deep wells in 18 mm diameter aluminum disks, with standards placed strategically so that the lateral neutron flux gradients across the disk could be evaluated. Planar regressions were fit to the standard data, and the $^{40}Ar/^{39}Ar$ neutron fluence parameter, J, interpolated for the unknowns. Uncertainties in J are estimated at 0.1-0.2% (1s), based on Monte Carlo error analysis of the planar regressions (Best et al., 1995). All specimens were irradiated in the cadmium-lined, in-core CLICIT facility of the Oregon State University TRIGA reactor. The duration of irradiation was 12 h and using the Fish Canyon sanidine (Kuiper et al., 2008) and GA1550 biotite (Spell and McDougall, 2003) standards.

Irradiated samples were placed in a Cu sample tray, with a KBr cover slip, in a stainless steel high vacuum extraction line and baked with an infrared lamp for 24 h. Selected sample volumes were fused using the laser, and reactive gases were removed, after ~3 min, by three GP-50 SAES getters (two at room temperature and one at 450°C) prior to being admitted to an ARGUS VI mass spectrometer by expansion. Five argon isotopes were measured simultaneously over a period of 6 min. Measured isotope abundances were corrected for extraction-line blanks,

which were determined before every sample analysis. Line blanks averaged ~5 fA for mass 40 and ~0.024 fA for mass 36.

Mass discrimination was monitored by online analysis of air pipettes. A value of 295.5 was used for the atmospheric $^{40}Ar/^{36}Ar$ ratio (Steiger and Jäger, 1977) for the purposes of routine measurement of mass spectrometer discrimination using air aliquots, and correction for atmospheric argon in the $^{40}Ar/^{39}Ar$ age calculation. Corrections are made for neutron-induced ^{40}Ar from potassium, ^{39}Ar and ^{36}Ar from calcium, and ^{36}Ar from chlorine (Roddick, 1983; Renne et al., 1998; Renne and Norman, 2001). Analytical results are reported in **Table S1**.

Figure S1. (next three pages) Geometric quality (left) and AVA (right) diagrams from the thin sections analyzed for quartz petrofabrics in this study. Quartz displays high quality (lighter shades of gray) in the geometric quality scans, while other mineral phases show significantly poorer quality (darker shades of grey). In sample EV21-07, isolated porphyroblasts of epidote also show an apparent high quality. Color wheels on the right of AVA diagrams show correspondence of grain color to mineral c-axis in a lower hemisphere projection.

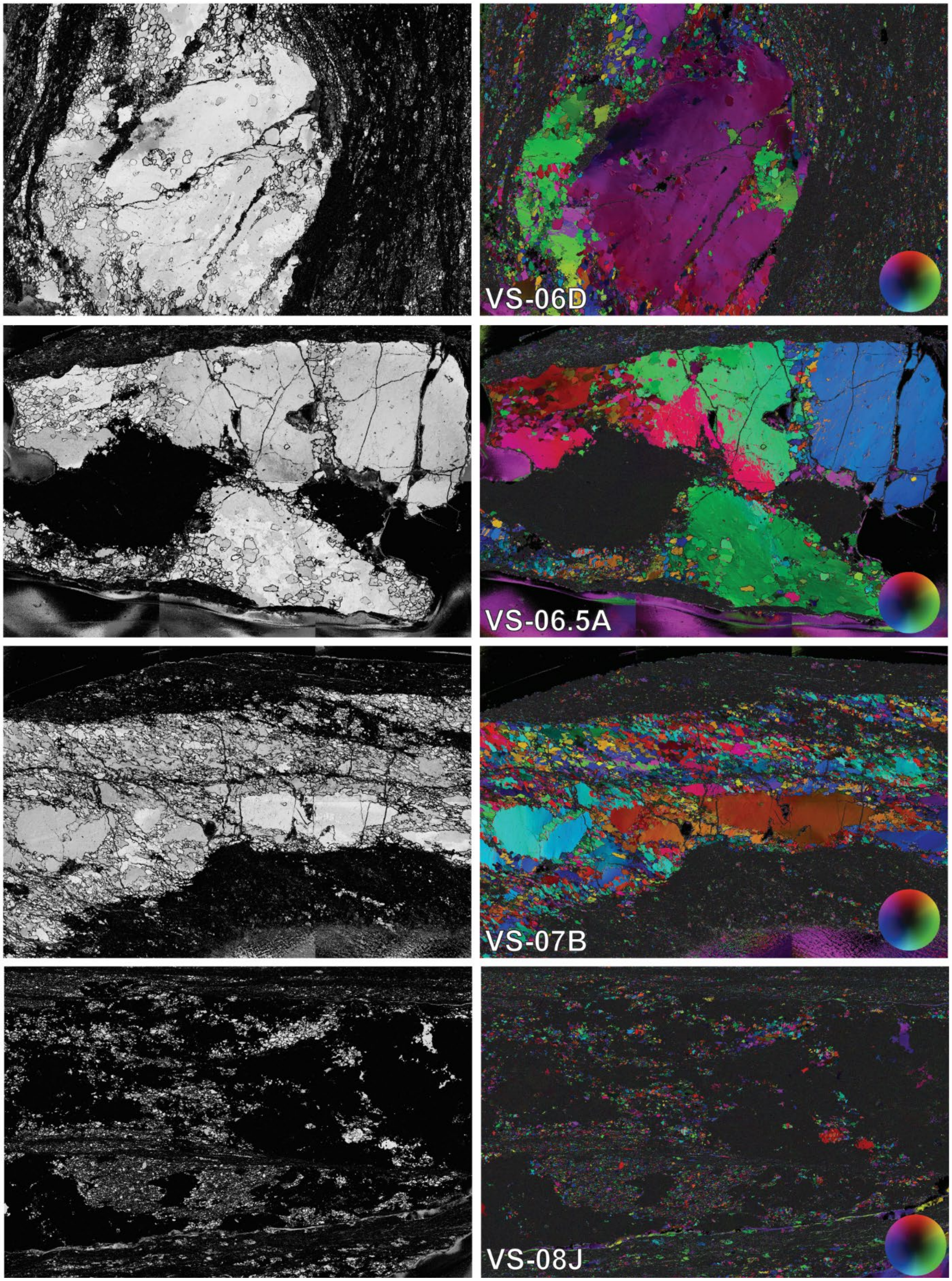

VS-06D
VS-06.5A
VS-07B
VS-08J

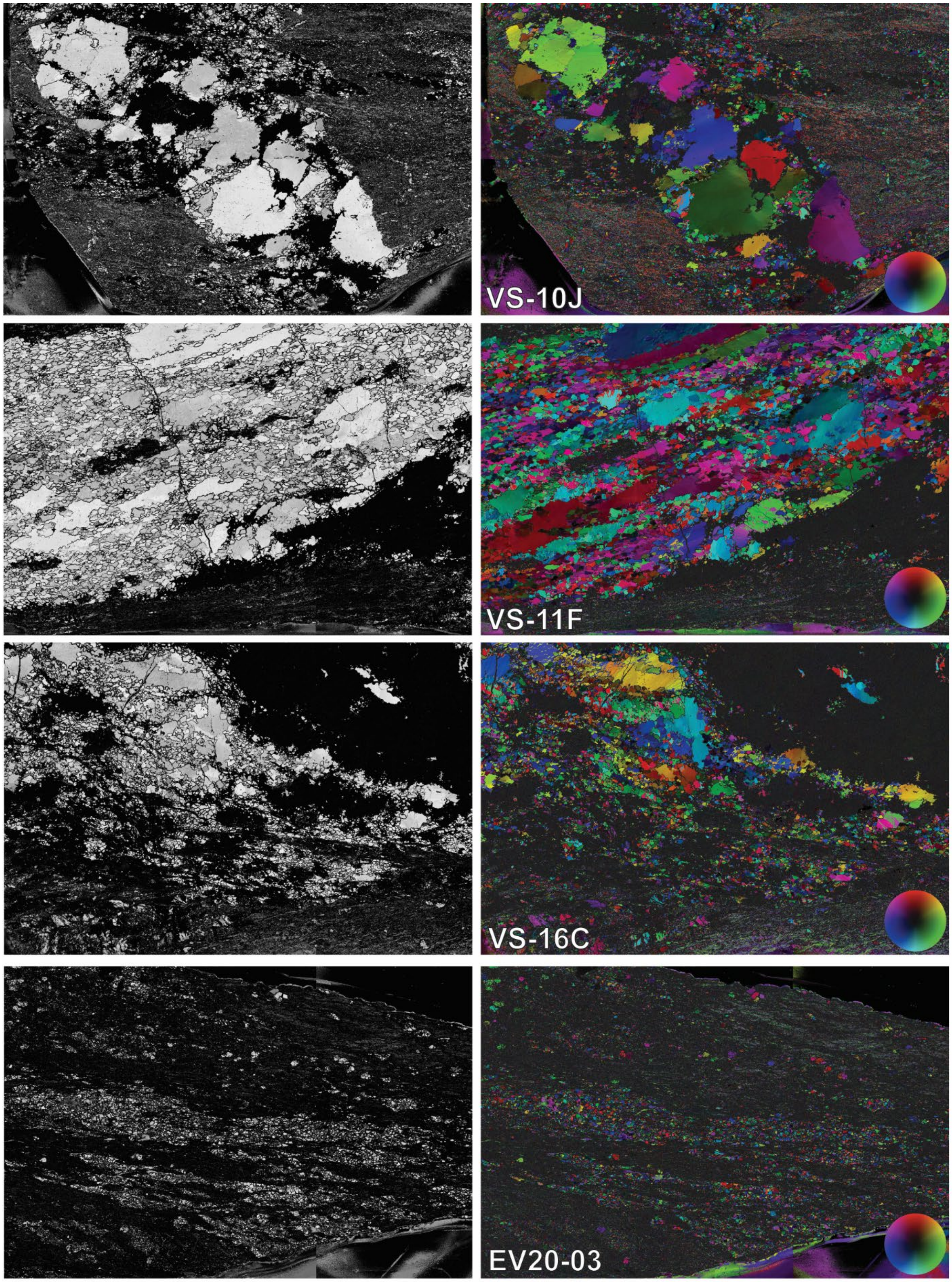
VS-10J
VS-11F
VS-16C
EV20-03

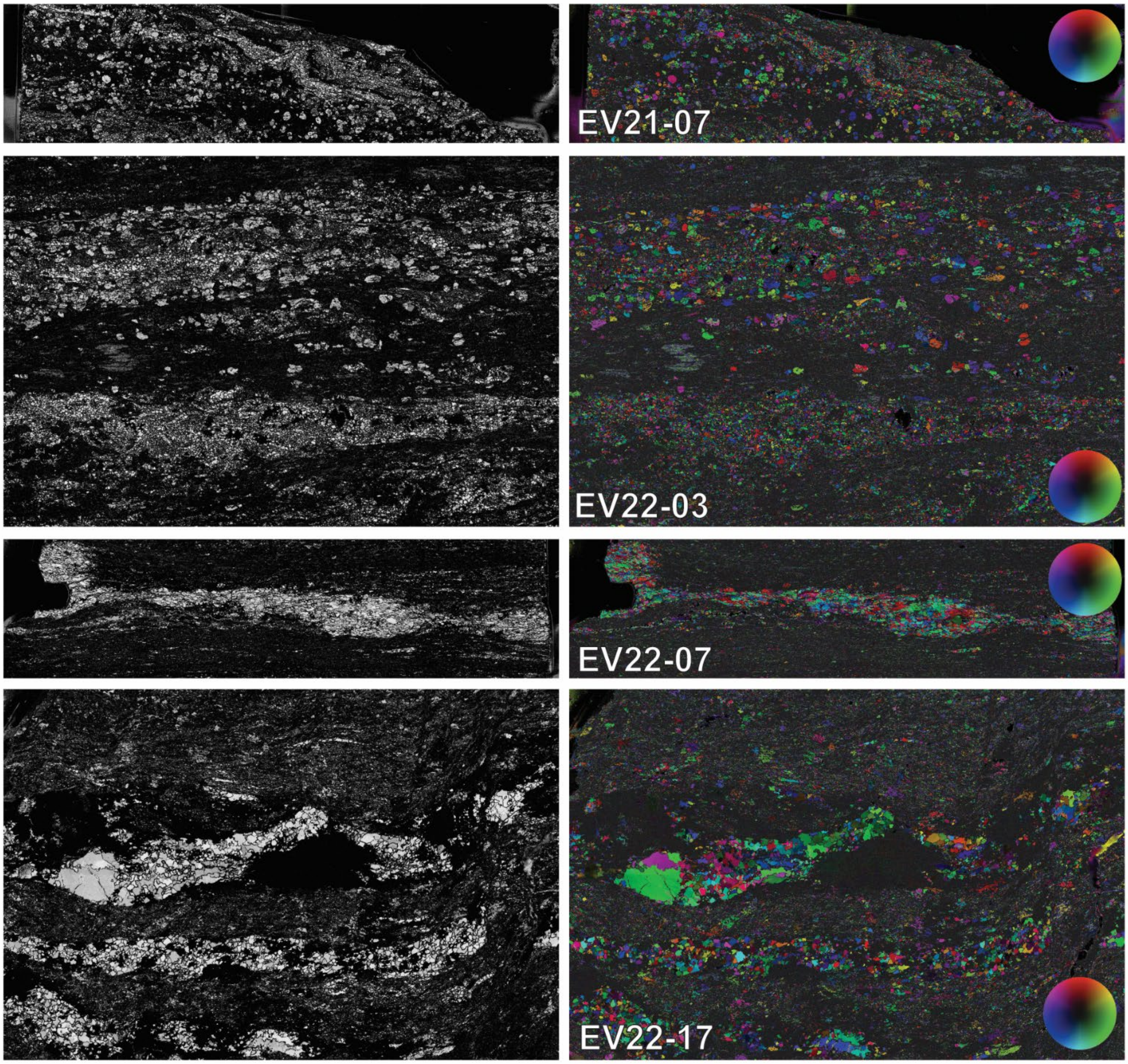
EV21-07
EV22-03
EV22-07
EV22-17

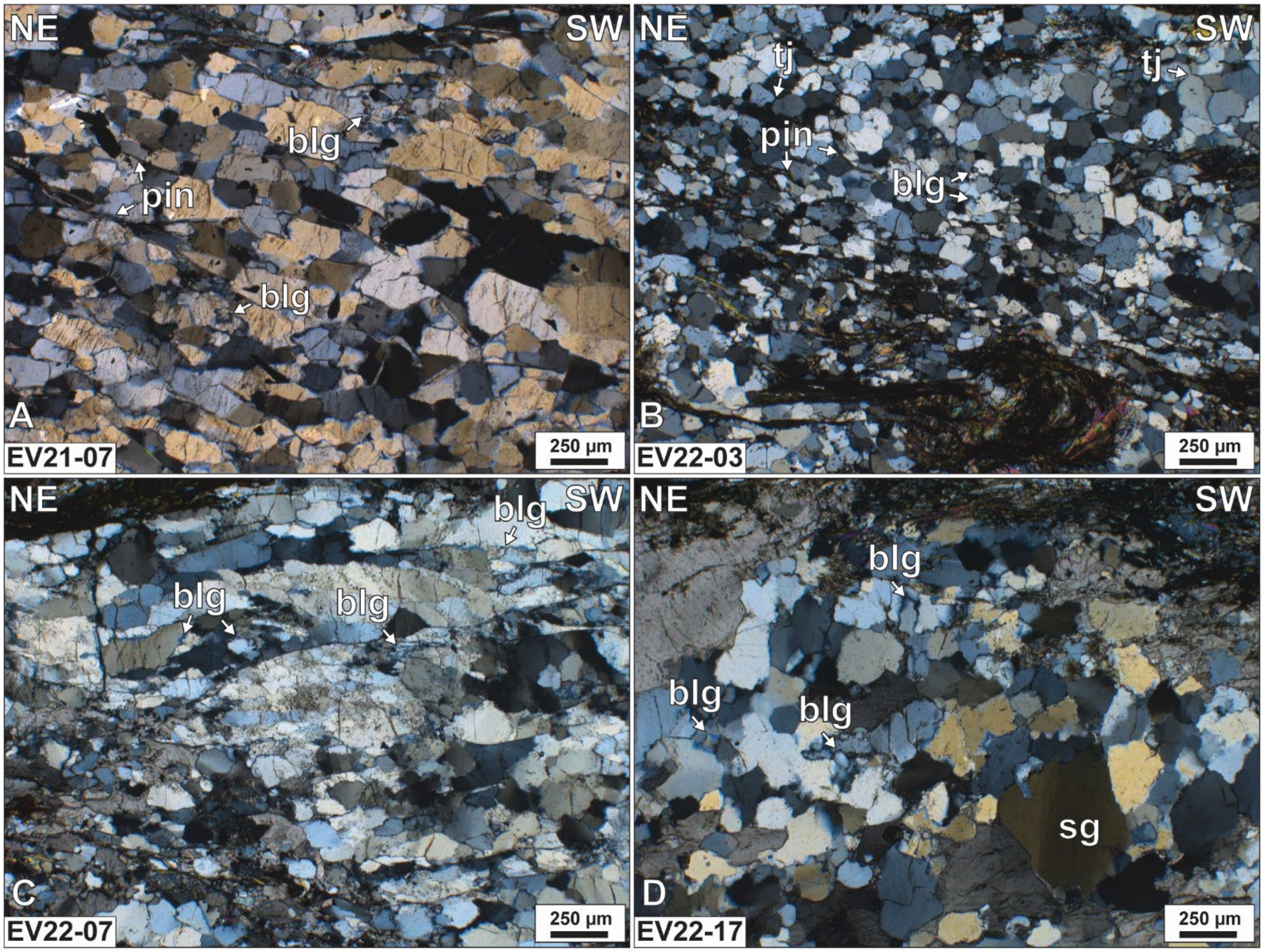

Figure S2. Photomicrographs of vein quartz samples selected for quartz c-axis fabric analysis from the Basal Unit flysch of southern Evia. (A) Sample EV21-07, situated at high structural levels. Blocky polygonal quartz aggregates with a moderately well-defined shape-preferred orientation (SPO) are distributed between pinning phyllosilicates. The blocky microstructure is locally overprinted by patches showing serrated and bulging grain boundaries. (B) Sample EV22-03, from high structural levels near the Mesochoria cataclasite. Fine polygonal aggregates with abundant 120° grain boundary triple junctions show minor local overprint by grain boundary bulging. (C) Sample EV22-07, from intermediate parts of the structural pile on southern Evia. Elongate parent grains, possibly remnants of recrystallized ribbon quartz, show overprinting by grain boundary bulging recrystallization that is more intense than observed elsewhere in the structural pile. (D) Sample EV22-17, from near the top of the structural pile. Planar subgraining is visible in larger quartz crystals and grain boundaries show sporadic bulging. Abbreviations: blg – grain boundary bulging recrystalliztation; pin – pinning; tj – grain boundary triple junction.

Table S1.
Electron microprobe mineral chemistry analyses of white mica used for *in situ* $^{40}Ar/^{39}Ar$ analysis from southern Evia (Greece).

Table S2.
Analytical results for *in situ* white mica $^{40}Ar/^{39}Ar$ geochronology for samples from southern Evia (Greece).

Table S3.
Analytical results for *in situ* white mica $^{87}Rb/^{87}Sr$ geochronology for samples from southern Evia (Greece).
